# An “eat me” combinatory nano-formulation for systemic immunotherapy of solid tumors

**DOI:** 10.7150/thno.56936

**Published:** 2021-08-11

**Authors:** Hend Mohamed Abdel-Bar, Adam A Walters, Yau Lim, Nadia Rouatbi, Yue Qin, Fatemeh Gheidari, Shunping Han, Rihab Osman, Julie Tzu-Wen Wang, Khuloud T. Al-Jamal

**Affiliations:** 1 Institute of Pharmaceutical Science, Faculty of Life Sciences & Medicine, King's College London, Franklin-Wilkins Building, 150 Stamford Street, London SE1 9NH, United Kingdom.; 2 Department of Pharmaceutics, Faculty of Pharmacy, University of Sadat City, P.O. box: 32958 Egypt.; 3 Faculty of Pharmacy-Ain Shams University, Abbassia, Cairo, P.O. box: 11566 Egypt.

**Keywords:** SNALPs, Immunogenic cell death, doxorubicin, CD47, calreticulin

## Abstract

**Rational:** Tumor immunogenic cell death (ICD), induced by certain chemotherapeutic drugs such as doxorubicin (Dox), is a form of apoptosis potentiating a protective immune response. One of the hallmarks of ICD is the translocation of calreticulin to the cell surface acting as an 'eat me' signal. This manuscript describes the development of a stable nucleic acid-lipid particles (SNALPs) formulation for the simultaneous delivery of ICD inducing drug (Dox) with small interfering RNA (siRNA) knocking down CD47 (siCD47), the dominant 'don't eat me' marker, for synergistic enhancement of ICD.

**Methods:** SNALPs loaded with Dox or siCD47 either mono or combinatory platforms were prepared by ethanol injection method. The proposed systems were characterized for particle size, surface charge, entrapment efficiency and *in vitro* drug release. The ability of the SNALPs to preserve the siRNA integrity in presence of serum and RNAse were assessed over 48 h. The *in vitro* cellular uptake and gene silencing of the prepared SNALPs was assessed in CT26 cells. The immunological responses of the SNALPs were defined *in vitro* in terms of surface calreticulin expression and macrophage-mediated phagocytosis induction. *In vivo* therapeutic studies were performed in CT26 bearing mice where the therapeutic outcomes were expressed as tumor volume, expression of CD4 and CD8 as well as *in vivo* silencing.

**Results:** The optimized SNALPs had a particle size 122 ±6 nm and an entrapment efficiency > 65% for both siRNA and Dox with improved serum stability. SNALPs were able to improve siRNA and Dox uptake in CT26 cells with enhanced cytotoxicity. siCD47 SNALPs were able to knockdown CD47 by approximately 70% with no interference from the presence of Dox. The siCD47 and Dox combination SNALPs were able to induce surface calreticulin expression leading to a synergistic effect on macrophage-mediated phagocytosis of treated cells. In a tumor challenge model, 50% of mice receiving siCD47 and Dox containing SNALPs were able to clear the tumor, while the remaining animals showed significantly lower tumor burden as compared to either monotreatment.

**Conclusion:** Therefore, the combination of siCD47 and Dox in a particulate system showed potent anti-tumor activity which merits further investigation in future clinical studies.

## Introduction

Selective cancer treatments, such as certain chemotherapeutics (anthracyclines, Oxaliplatin) and radiotherapy, stimulate cells to undergo immunogenic cell death (ICD) 1. ICD is a defined type of apoptosis, first described by the works of Kroemer and Zitvogel, which potentiates an inflammatory immune response 2. For instance, it has been shown that tumor cell lines treated with ICD inducer then implanted into mice serve to 'vaccinate' mice from re-challenge with homologous, but not heterologous, tumor cells 3. Furthermore, that established tumors treated locally with high dose ICD can cause remission of distal tumors, suggesting the establishment of systemic immunity 4. There are many defining physiological characteristics of ICD, these being: the expression of calreticulin on the cell surface, the release of damage-associated molecular patterns (DAMPs) such as adenosine triphosphate (ATP) and heat shock proteins (HSPs) and the release of high mobility group box 1 (HMGB1) 5. During ICD: surface calreticulin acts as an 'eat me' signal engaging with low-density lipoprotein receptor-related protein 1 (CD91) on phagocytes, the release of DAMPs delivers a 'find me' signal and HMGB1 causes maturation of antigen-presenting cells (APCs) through interaction with Toll-like receptor 4 (TLR4) 6. It is a combination of these factors which lead to the activation of the immune system 7. Of these factors, the surface expression of calreticulin is believed to be the single most essential element for bona fide ICD 8. Indeed, the propensity of drugs to cause cells to undergo ICD can be correlated with surface calreticulin levels 9. However, while ICD is extremely potent, the effect of calreticulin exposure is believed to be counterbalanced, and potentially dampened, by CD47 expression 10.

CD47 is widely expressed and, after engagement with SIRPα present on phagocytes, serves to deliver a 'don't eat me' signal 11. Tumors co-opt this axis by highly expressing CD47 to escape clearance by the mononuclear phagocytic system 12. It has been suggested that even in the presence of surface calreticulin, CD47 may still inhibit phagocytosis, therefore blocking this interaction while delivering ICD might enhance the effect 13. Blocking CD47 has previously been attempted using multiple modalities including conventional monoclonal antibodies and siRNA, of which antibody-based systems are the most advanced having reached clinical trials 14. It has been suggested that antibodies may be ideally suited to this purpose as, in addition to blocking the CD47 axis, there will be an interaction between the antibody constant region and Fc receptors serving to further aid uptake of cancer cells by phagocytic cells 15.

However, it could also be speculated, that due to CD47's broad expression, a tumor-targeted delivery of the inhibitors could be more suitable. In this regard, targeting serves not only to ensure the agent is concentrated at the tumor site, but will also limit dilution through off-target interaction. As a corollary of this, systemic side effects should also be negated. Tumor targeting may be difficult to achieve with the conventional monoclonal antibody system as it would require the antibody to be formulated or engineered to be tumor specific. To this end, the utilization of targeted siRNA against CD47 may be advantageous. siRNA is easy to manufacture and modify and it can be, relatively simply, formulated into nanocarriers capable of targeting the tumor through the enhanced permeation and retention effect (EPR) 16. Indeed, previous data have shown that CD47 siRNA nanoparticles are able to induce a significant delay or inhibition of tumor growth in isolation or combined with knockdown of other targets 17. In addition to this, it may be speculated, formulation in a particulate system allows for the co-delivery of siRNA and ICD inducer in a single vector ensuring a spatio-temporal relationship between the initiation of ICD and knockdown of CD47 is established. While there have been several recent studies showing anti CD47 therapy and ICD induction results in synergistic efficacy 18, 19, to our knowledge this synergistic effect has never been demonstrated in a siRNA-based approach. Furthermore, the means to deliver siCD47 and Dox have never been validated. With siRNA approaches showing to be highly efficacious in pre-clinical models 17, 20, it is important to develop a means of drug delivery and to establish whether this effect is maintained prior to potential clinical evaluation.

There are intrinsic obstacles to the delivery of siRNA such as degradation in the bloodstream which diminishes therapeutic efficacy. To circumvent this, the stable nucleic acid-lipid nanoparticle (SNALPs) platform was developed 21. Since its invention, the SNALPs platform has become the dominant means to deliver RNA in the literature, due to several factors such as: ease of manufacture, scalability, minimal toxicity and potency 22. SNALPs contain a mixture of cationic lipids and fusogenic lipids, enabling high encapsulation efficiency of nucleic acids with improved cellular uptake and subsequent release 23. The inclusion of polyethylene glycol (PEG)-lipids coating not only stabilizes particles but also favors their *in vivo* application, preventing rapid systemic clearance after systemic injection 22. In addition, SNALPs have a high surface-to-volume ratio so can deliver a large quantity of materials, can be engineered to include cytotoxic drugs, such as ICD inducers, and are not limited by tissue tropism or safety concerns as is the case for more traditional means of delivery such as viral vectors.

This study investigates the development of a SNALPs based system for co-delivery of ICD inducing drug (doxorubicin) and siRNA, to knockdown CD47, with the aim to synergistically improve tumor survival. Herein, we optimized SNALPs loaded with doxorubicin and siRNA with a particle size less than 200 nm and maximum entrapment efficiency for both doxorubicin and siRNA. The ability of the developed SNALPs to improve the cellular uptake of doxorubicin as well as siRNA in CT26 cells was investigated. The effect of the prepared SNALPs on calreticulin expression and macrophage uptake was studied. The therapeutic efficacy of the SNALPs loaded with doxorubicin and siCD47 on CT26 cells was explored in comparison to each drug alone to prove the hypothesis.

## Materials

1, 2-distearoyl-snglycero-3-phosphocholine (DSPC), 1, 2-dioleoyl-3-trimethylammonium-propane (DOTAP), N-palmitoyl-sphingosine-1-[succinyl (methoxypolyethylene glycol) 2000] (C16-PEG2000 Ceramide) were purchased from Avanti Polar Lipids (USA). Cholesterol (CH), 1,1'-Dioctadecyl-3,3,3',3'-tetramethylindotricarbocyanine Iodide (DiR), dialysis tubing (MWCO 12 kDa), absolute ethanol, dimethylsulphoxide (DMSO), Triton X-100 were supplied from Sigma-Aldrich, UK. RPMI-1640 media, fetal calf serum (FCS), penicillin/streptomycin, Trypsin/EDTA, and phosphate buffered saline (PBS) were obtained from Gibco, Invitrogen (UK). Formaldehyde was from Thermo Scientific Pierce, UK. Isoflurane (IsoFlo®) for anaesthesia was purchased from Abbott Laboratories Ltd, UK. All reagents were used without further purification. ATP assay (CellTitre Glo) was purchased from Promega (UK). CD47 siRNA was purchased from Dharmacon (UK), Doxorubicin was purchased from Apollo Scientific (UK) and Cisplatin was obtained from QILU Pharmaceutical Co. Ltd (China). Anti-mouse CD8-PE clone 53-6.7, Anti-mouse CD4-FITC clone GK1.5, Anti-mouse Interferon gamma-APC clone XMG1.2, Anti-mouse CD47-APC clone miap301, Anti-rabbit IgG-FITC clone Poly4064 were purchased from Biolegend. Anti-Human Calreticulin clone Ab2907 was purchased from Abcam.

## Methods

### Preparation of SNALPs

SNALPs formulations with or without Dox were prepared using ethanol injection method as described elsewhere with slight modifications 24. For *in vitro* studies, a lipid mixture was prepared from CH: DSPC: DOTAP: C16-PEG2000-Ceramide, with different molar ratios at final lipid amount of 0.213 µmole (**Table S1**), in absolute ethanol at 60 °C (40 µl). Aqueous solution of siRNA (1 nmole) was diluted with 20 mM citrate buffer (60 µl pH 4, in RNAse free water) and heated at 60 °C. The aqueous siRNA solution was titrated with the alcoholic lipid solution dropwise under vigorous vortex to ensure the formation of SNALPs. The obtained SNALPs were incubated at 40 °C for 1 h. The unentrapped siRNA was removed by ultrafiltration centrifugation using MWCO 100K at 14,000 rpm for 45 min and the prepared SNALPs were re-dispersed in HEPES buffer (to final volume of 100 µl pH 7, in RNAse free water). Different Dox loaded SNALPs were prepared using pH gradient method 25. The SNALPs suspension pH was raised from pH 4 to pH 8 using 0.5 M sodium bicarbonate and heated at 60 °C for 5 min. Subsequently, the SNALPs were incubated with preheated equivolume of Dox solution (in PBS pH 7.4 at drug to lipid ratio 0.2: 1 w/w) at the same temperature for 2 h. Buffer exchange was then performed for all SNALPs (with/or without Dox) using ultrafiltration (MW CO 30K at 14,000 rpm, 45 min) to replace the external buffer with HEPES buffer (pH 7) and to remove the unentrapped payload. Lipid, siRNA and Dox amounts for formulations used in *in vivo* imaging and therapy studies are summarized in **Table S2**. For DiR-labeled SNALPs_siNeg_ used in the biodistribution study, DiR was incorporated in at 1 mole % of total lipid.

### Determination of particle size, size distribution and zeta potential

The particle size (z-average), charge and size distribution expressed as polydispersity index (PDI) of the prepared SNALPs (+/-Dox) were estimated by using dynamic light scattering (DLS) with a Nanosizer ZS Series (Malvern Instruments, Southborough, MA). Briefly, different SNALPs were diluted with deionized water (1:10 v/v) and transferred to disposable plain folded capillary Zeta cells. All the measurements represent the average of 20 runs, each run was completed in triplicate at 25 °C.

### Determination of Encapsulation Efficiency (EE %)

The encapsulation efficiency (EE %) of both siRNA or Dox was quantified indirectly by measuring the difference between the total amount of siRNA or Dox added during the preparation of SNALPs and the quantity of non-entrapped amounts remaining in the filtrate after the SNALPs were buffer exchanged. The siRNA amount was determined using gel red assay technique using previously constructed calibration curve. In brief: to construct the calibration curve, different volumes of siRNA were mixed with gel red (3 µl, 1:1000) then the volume was adjusted to 100 µl with Tris buffer pH 7. The coefficient of determination (R^2^) of the siRNA calibration curve in Tris buffer pH 7 in the concentration range of 0.1-1 nM, was 0.9977 **(Figure S1A)**. The amount of siRNA was determined using UV-vis spectrofluorometer at excitation and emission of 300 and 590 nm respectively (Model UV-1601 PC; Shimadzu, Kyoto, Japan). The amount of Dox was determined using previously constructed standard plot of serial concentrations of Dox in PBS pH 7.4 by measuring the absorbance at 480 nm as reported in the literature 26 **(Figure S1B)**. The EE % of either siRNA or Dox was calculated as follows:

EE (%) = (([Drug] total - [Drug] free)/ [Drug] total) *100

### Gel retardation assay

The efficiency of the fabricated SNALPs to load siRNA was evaluated qualitatively using gel electrophoresis. Moreover, the ability of SNALPs to protect the encapsulated siRNA integrity against FCS (10 and 50% v/v) and RNAse (100 µg/mL) was evaluated after heparin competition assay. SNALPs_siNeg_ (1 nmole, 100 µl) were incubated with FCS or RNAse for 4, 24 and 48 h at 37 °C. Following incubation, EDTA, to a final concentration of 50 mM, was added to inhibit the RNAse activity. Heparin was added to a final concentration of 10% v/v (100 IU/mL) to dissociate the SNALPs complex. Naked siRNAs which had been incubated either with FCS, RNAse or left untreated were used as a positive and negative control respectively. The samples (5 µL) were mixed with 6x loading dye and analyzed using electrophoresis on agarose gel (2 % w/v agarose in sodium borate buffer, 225 V for 20 mins). Bands were visualized under UV light (MP system, BioRad, UK) after being stained with Gel red. In the case of serum co-incubated SNALPs, a sample was taken and analyzed for size, PDI and zeta potential.

### Transmission electron microscope

The sample was placed on 300-mesh carbon-coated copper grids and left to air-dry. Negative staining was achieved using 3% uranyl acetate for 3 min followed by washing once with water and left to air-dry. The grids were imaged by a Philips CM 12 transmission electron microscope (FEI Electron Optics, The Netherlands) equipped with Tungsten filament and a Veleta - 2k × 2k side-mounted TEM CCD camera (Olympus, Japan) at the accelerating voltage of 80 kV.

### *In vitro* doxorubicin release

SNALPs_siNeg-Dox_ containing Dox equivalent to 1 mg were sealed into 12 kDa dialysis membrane. The SNALPs (3 mL) with or without FCS (50% v/v) were dialyzed against PBS (10 mL), pH 7.4. To study the effect of pH on Dox release, SNALPs_siNeg-Dox_ were dialyzed against acetate buffer pH 5.5 (10 mL, pH 5.5) at 37 °C. For comparison, and to eliminate nonspecific drug binding to the dialysis membrane, *in vitro* release of Dox was also performed in acetate buffer pH 5.5. At predetermined time intervals, an aliquot of 1 mL of the dialysate was withdrawn and replenished by fresh preheated media. Drug concentration was quantified by measuring the absorbance at 480 nm using a UV spectrophotometer (Perkin-Elmer Lambda 35) using plain SNALPs as a blank. Dox concentration was calculated using pre-constructed calibration curves in PBS pH 7.4 or acetate buffer 5.5 with a respective R^2^ of 0.9968 and 0.9972 in the range of 2 and 10 μg/mL (**Figure S1B and C**).

### Hemolysis test

Haemolytic activity of Dox either free or encapsulated in SNALPs were assessed using fresh BALB/c red blood cells (RBCs). Briefly, 1 mL blood was withdrawn from BALB/c by cardiac puncture into heparinized tube and the blood was centrifuged at 4000 rpm for 10 min. The obtained RBCs were incubated with serial concentrations of Dox (0-60 nM) in PBS pH 7.4 for 2 h at 37 °C. Subsequently, the samples were centrifuged at 4000 rpm for 5 min at 4 °C. The absorbance of each supernatant was determined at 545 nm. Fresh RBCs incubated with 0.5% v/v Triton X-100 or PBS pH 7.4 were used as a positive and negative control, respectively. The % hemolysis was calculated using the following equation:


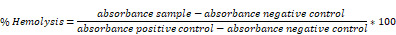


### Cell culture

CT26 murine colon carcinoma cells were cultured in RPMI media supplemented with 10% v/v FCS, 50 U/mL penicillin, 50 µg/mL streptomycin and 1% v/v L-glutamine. Cells were incubated in 5% CO_2_ at 37 °C.

### Cellular uptake studies by flow cytometry

Dox uptake and/ or siRNA were detected using flow cytometry. Confluent CT26 cells were treated with three different concentrations of Dox (10, 30 and 60 nM) either in solution form or SNALPs_siNeg-Dox_. The ability of SNALPs to improve the uptake of siRNA was also performed using fluorescently labelled siRNA (Atto 655-siRNA) at a concentration of 10, 30 and 90 nM. The effect of Dox on Atto 655-siRNA uptake was conducted using a co-incubation of SNALPs_siAtto 655-Dox_ at a serial concentration of Dox (10, 30 and 60 nM) on Atto 655-siRNA (30 nM) uptake. After treatment and incubation of 4, 6, 8 or 24 h, the cells were washed twice with PBS, trypsinized and centrifuged at 1750 rpm for 3 min. The cellular uptake of Dox or Atto 655-siRNA either soluble or encapsulated in SNALPs was studied on 10 k gated cells by quantifying the fluorescence using FL3 and FL4 detector, respectively (BD FACS Calibur™ flow cytometer, BD Biosciences). Analysis was performed using Flowjo^TM^ software (Treestar). When using both Atto 655 and Dox, the quenching effect of Dox was compensated (see supplementary methods **Figure S2**).

### Cytotoxicity study

CT26 cells in 96-well (7 k cells/ well) in RPMI media were incubated with serial dilutions of Dox (solution or as SNALPs) ranging from 0.01-100 µM. Cells were also incubated with SNALPs free from Dox and/ or DOTAP in the same dilutions to eliminate the toxicity of the vehicle. *In vitro* cytotoxicity was examined by MTT assay. Briefly, after 48 h incubation media was aspirated and replaced with 120 μl of MTT solution then cells were incubated for 4 h at 37 °C and 5% CO_2_. The formed formazan crystals were dissolved in 200 μL of DMSO then the plate was read at 570 nm using FLUO star OPTIMA plate reader (BMG Labtech). The results were expressed as the percentage cell survival which was calculated using the following equation:

Cell survival (%) = (A570 nm of treated cells/A570 nm of untreated control cells) × 100

### *In vitro* gene silencing

To assess the gene silencing effect, CT26 cells were incubated with SNALPs_siCD47_ (**Table S2**) at a concentration of 10, 30 and 90 nM for 48 h and 72 h. Consequently, media was aspirated, and cells were washed twice with PBS pH 7.4 and harvested by trypsin-EDTA. The cells were stained with anti-mouse CD47-APC monoclonal antibody and the expression of CD47 was quantified using the fluorescence in FL4 (BD FACS Calibur™ flow cytometer, BD Biosciences) after having been gated on FSC/SSC profile using Flowjo^TM^ software (Treestar). The influence of different Dox concentrations (10, 30 and 60 nM) on the gene silencing of CD47 were conducted by incubating CT26 with SNALPs_siCD47-Dox_ at a fixed concentration of siCD47 (30 nM) for 48 h. Cells were then harvested and analyzed as described above.

### Effect of doxorubicin on calreticulin expression

CT26 cells were incubated with Dox or SNALPs_siNeg-Dox_ (5, 10 and 20 µM) for 4 h in RPMI media as reported previously 27. Cells were incubated with matched concentrations of cisplatin solution as a negative control. Media was removed, and the cells were washed with PBS pH 7.4 twice and collected by trypsin-EDTA solution. Cells were stained with rabbit anti-human calreticulin antibody followed by donkey anti-rabbit IgG-FITC secondary antibody and the fluorescence was measured by flow cytometry by first gating on FSC/SSC profile then assessing signal in FL1 channel. As a negative control, cells were also stained using secondary antibody only. The effect of siCD47 on calreticulin expression was investigated by incubating CT26 cells by SNALPs_siCD47-Dox_ containing 30 nM siCD47 and 5 µM Dox concentrations for 48 h to allow for gene silencing.

### Detection of acellular ATP

CT26 cells were either left untreated or exposed to SNALPs_siNeg-Dox_ at 30 nM and 60 nM for siNeg and Dox concentration, respectively. Following 24 h incubation cell supernatant was harvested. Supernatant was cleared of cellular components by centrifugation 3000xg, 5 mins. ATP was quantified using commercial luciferase-based assay in accordance with the manufacturer's specification. Light emission was detected using FLUO star OPTIMA plate reader (BMG Labtech).

### Effect of co-culture of CT26 murine colon cancer cells by different SNALPs on macrophage uptake

To generate 'target cells', CT26 cells were pulsed with either: SNALPs_siNeg_, SNALPs_siNeg-Dox_, SNALPs_siCD47_ or SNALPs_siCD47-Dox_ at 30nM siCD47 and 60nM Dox for 48 h_._ Cells were pre-stained with Cell Trace^TM^ (Invitrogen) as described in the manufacturer instructions. Treated and labelled CT26 were collected by trypsin-EDTA solution then added to J774 macrophage cells for 6 h. The J774 cells were harvested and stained with fluorophore conjugated anti mouse CD45 monoclonal antibody to differentiate J774 from CT26. Cells were acquired using a FACs Calibur^TM^ flow cytometer and data analyzed using Flowjo^TM^ (Treestar). Macrophage uptake was determined by first gating on CD45+ population prior to measuring CellTrace^TM^ MFI within this population.

### *In vivo* imaging and organ biodistribution

All animal experiments were conducted in agreement with the personal licenses granted by the UK Home Office and in accordance with the UKCCCR Guidelines (1998). BALB/c mice 4-6 weeks (Envigo) were inoculated with CT26 cells (1x10^6^ cells/mouse in PBS) by subcutaneous (SC) injection in one side of the lower flank. Three mice were intravenously injected with 200 µl DiR-labeled SNALPs_siNeg_ in HEPES buffer pH 7 containing approximately 21.99 mg/kg, 0.86 mg/kg and 0.22 mg/kg for total lipid, siRNA and DiR respectively (**Table S2**). DiR fluorescence dye was incorporated during the formulation of SNALPs at 1 mole % of the total lipid. The anesthetized animals were imaged immediately after injection and at 1, 4 and 24 h post-injection using an IVIS Lumina Series III *In vivo* Imaging System (Caliper Life Sciences, Perkin Elmer, USA) using excitation/emission wavelengths 750/780 nm with exposure time 5 sec. Untreated animals were used as a negative control to eliminate auto-fluorescence. Animals were culled at 24 h post-injection and their vital organs (brain, heart, lung, liver, spleen and kidneys), as well as the tumor, were excised and weighed for analysis.

### *In vivo* therapeutic study

To determine the therapeutic efficiency of the fabricated systems on CT26 tumor growth, the tumor bearing mice were randomly divided into 4 groups (n = 10). Animals were anesthetized using isoflurane and injected intravenously with either PBS (pH 7.4), SNALPs_siNeg-Dox,_ SNALPs_siCD47_ or SNALPs_siCD47-Dox_ with lipid, siCD47 and Dox dose of 36.23 mg/kg, 0.1 mg/kg and 5 mg/kg respectively (**Table S2**). The study was conducted over 32 days with two injections at days 7 and 17 post tumor inoculation. Tumor volume and change in body weight were monitored for each animal 3-4 times per week. At the end of the study, serum was collected from all mice then animals were sacrificed by cervical dislocation. Tumor and the tumor draining lymph nodes were extracted for further assessment and the major organs including heart, lung, kidney, liver and spleen were collected and weighed.

### Immunological assessment following tumor challenge

Spleens and tumors were extracted postmortem. Single cell suspensions from each organ were obtained by physical dissociation of tissue with cell strainer. Splenocytes were cultured in RPMI media supplemented with 10% FCS, 50 U/mL penicillin, 50 µg/mL streptomycin and 1% L-glutamine. Cells were incubated in 5% CO_2_ at 37 °C in the presence of 1× Brefeldin A (Biolegend) for 4 h. Cells were harvested, and surface markers were stained with anti-mouse CD4-FITC and anti-mouse CD8α-PE monoclonal antibodies prior to washing and fixation with 4% paraformaldehyde. Cells were permeabilized with 1x intracellular staining permeabilization buffer (Biolegend) in accordance with the manufacturer's protocol. Intracellular IFNγ was stained with anti-mouse IFNγ-APC before being washed and acquired on a FACs Calibur^TM^ flow cytometer. Cells were first gated on FSC/SSC before specific marker was analyzed. Cells extracted from tumors were stained with anti-mouse CD4-FITC and anti-mouse CD8α-PE monoclonal antibodies and acquired on a FACs Calibur^TM^ flow cytometer. Absolute cell number was calculated from normalizing the number of events to acquisition volume.

### Immunohistochemistry & staining analyses

Sections (8 μm) were cut from formalin-fixed paraffin-embedded xenograft tumors for immunohistochemistry. Sections were deparaffinized in xylene and rehydrated in graded series of ethanol (100%, 95%, 70%). Endogenous peroxidase was quenched in 0.7% v/v hydrogen peroxide in methanol. Antigen retrieval (AR) by heating in citrate acid-based antigen unmasking solution (Vector Laboratories, H-3300) in the microwave for 15 min (HIAR) or incubation with proteinase K (New England Biolabs, P8107S) in Tris-buffered saline (TBS) with 0.1% v/v Tween 20 (TBS-T 0.1%) for 15 min at room temperature (RT) was performed depending on the antibody used as indicated. Non-specific binding was blocked with 10% v/v normal goat serum (NGS; Vector Laboratories, S-1000) in TBS with 0.25% v/v Triton X-100 (TBS-Tx 0.25%). Primary antibodies, rabbit anti-CD3, clone SP7 (HIAR, 1:1000, Abcam, ab16669) or rat anti-F4/80, clone Cl:A3-1 (Proteinase K, 1:100, Bio-Rad, MCA497) with 1% v/v NGS in TBS with 0.1% v/v Triton X-100 (TBS-Tx 0.1%) were incubated on sections overnight at 4 °C. Biotinylated goat anti-rabbit IgG (1:200, Vector Laboratories, BA-1000) or biotinylated goat anti-rat IgG (1:100, Vector Laboratories, BA-9401) in TBS-T 0.1% were incubated on sections for 1 h at RT. Sections were visualized with VECTASTAIN Elite ABC-HRP Kit (Vector Laboratories, PK-6100) and 3,3'-diaminobenzidine tetrahydrochloride (DAB) Peroxidase (HRP) Substrate Kit (Vector Laboratories, SK-4100), followed by counterstaining in Mayer's hematoxylin (Sigma-Aldrich, MHS16-500ML). Sections were dehydrated in graded series of ethanol (70%, 95%, 100%) and cleared with xylene. Sections were cover slipped with DPX mounting medium. Slides were scanned using the VS120 Virtual Slide Microscope system (Olympus). Whole tumor sections at 20× objective magnifications were imported into ImageJ using the OlympusViewer Plugin.

CD47 immunohistochemistry staining was carried out using a Ventana Discovery automated staining instruments (ROCHE Ventana Medical Systems) following the manufacturer's guidelines, anti-CD47 (1:200 dilution, ab175388, Abcam), swine anti-rabbit IgG (1:200 dilution), horseradish peroxidase-conjugated streptavidin complex and diaminobenzidine as a chromogen. Stained sections were scanned on a Hamamatsu Nanozoomer S360 and CD47 staining was quantified using QuPath 28. Tumor areas were outlined as regions of interest (ROI). Necrotic and fibrous regions were excluded from the ROIs. A machine learning-based pixel classifier was trained to identify DAB staining in the ROIs. The area percentage of DAB staining in the ROIs were calculated and the mean DAB intensity measured.

### Histological examination of major organs

At the end of the therapy studies, major organs (i.e. heart, lung, liver, spleen and kidney) and tumors from different treatment groups were excised and fixed in 10% neutral buffer formalin. Tissues were paraffin-embedded and sectioned for hematoxylin and eosin stains (H&E) according to standard histological protocols at the Royal Veterinary College. All stained sections were imaged using a Leica DM 1000 LED Microscope (Leica Microsystems, UK) coupled with a digital camera (QImaging, UK).

### Statistical analysis

All the *in vitro* studies were done in three independent experiments and the data were the average of three measurements ±SD. For therapy experiments, data were presented as mean of 10 replicates ± SEM. Student T-test or Mann Whitney test was applied to compare two variables while ANOVA test followed by Tukey HSD test was used for comparing different parameters between groups. The distribution of data was first assessed by Shapiro Wilk normality test prior to statistical analysis is performed. Differences were considered statistically significant at probability (p) value less than 0.05.

## Results

### Formulation of SNALPs based siRNA chemotherapy co-delivery system

**Scheme 1:**
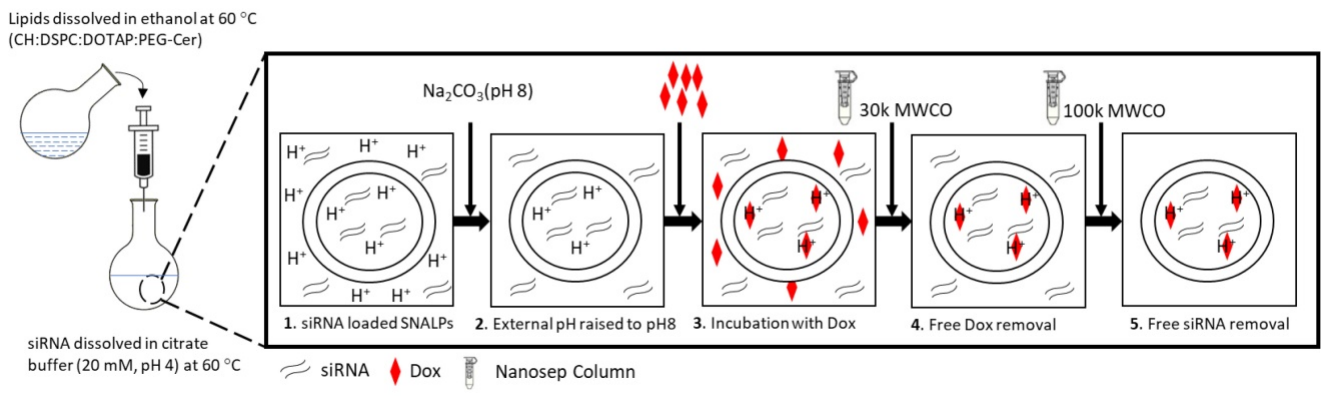
** SNALPs formulation process.** A lipid mixture of CH: DSPC: DOTAP: C16-PEG2000-Ceramide with different molar ratios were dissolved in absolute ethanol at 60 °C. Stock siRNA solutions in H_2_O were diluted in 20 mM citrate buffer, pH 4 and heated at 60 °C. The siRNA solution was titrated by the alcoholic lipid solution dropwise under vigorous vortex to ensure the formation of the SNALPs. The obtained SNALPs were incubated at 40 °C for 1 h. For Dox loaded SNALPs, sodium carbonate (1M) was added to adjust the external buffer to pH8 then the SNALPs were incubated with doxorubicin (20% w/w of total lipid) for 2 h at 60 °C. Buffer exchange was then be carried out using ultrafiltration (14,000 g, 45 min) to replace with HEPES buffer at pH 7 for SNALPs with or without doxorubicin.

**Scheme 1** illustrates the fabrication process of SNALPs with/without Dox. SNALPs of differing lipid composition were fabricated to obtain nanocarriers of less than 200 nm in size and with a maximum EE% for both siRNA and Dox **(Table S1)**. The particle size, expressed as z-average, ranged from 107.66 ±3.21 to 158.33±5.51 nm and followed the trend: Dox loaded lipid nanoparticles < siRNA loaded SNALPs < Dox-siRNA loaded SNALPs. For each preparation, the PDI was ≤ 0.2 indicating a monodisperse system was obtained. Due to the presence of DOTAP, all formulations were positively charged with a zeta potential between 5.23±0.42 and 11.13± 1.11 mV. In terms of loading, siRNA could be successfully encapsulated either alone or in the presence of Dox with efficiencies ranging from 47.11±3.98 to 65.11±6.25% **(Table S1)**. The ability of SNALPs to incorporate siRNA was confirmed by agarose gel electrophoresis **(Figure S3A)**. Only the fraction of the unentrapped siRNA into SNALPs could be detected in gel electrophoresis before ultrafiltration centrifugation of the SNALPs. On the contrary, after SNALPs purification, siRNA could not migrate on the gel electrophoresis due to the complexation with the aforementioned SNALPs. Likewise, Dox was incorporated to a high degree (> 80%). **Table S1** showed the EE% of Dox was significantly decreased in the presence of siRNA in all tested formulae (p < 0.05). Due to its size and loading, formulation F3 **(Table 1)**, hereafter referred to as 'SNALPs', was selected to progress to further studies. SNALPs containing: nonspecific negative siRNA (siNeg), Dox, Atto 655 labelled siRNA (siAtto655), siRNA targeting CD47 (siCD47) or a combination of the aforementioned will be labelled accordingly in the subscript.

### Biocompatibility and Biostability of SNALPs

**Figure 1:**
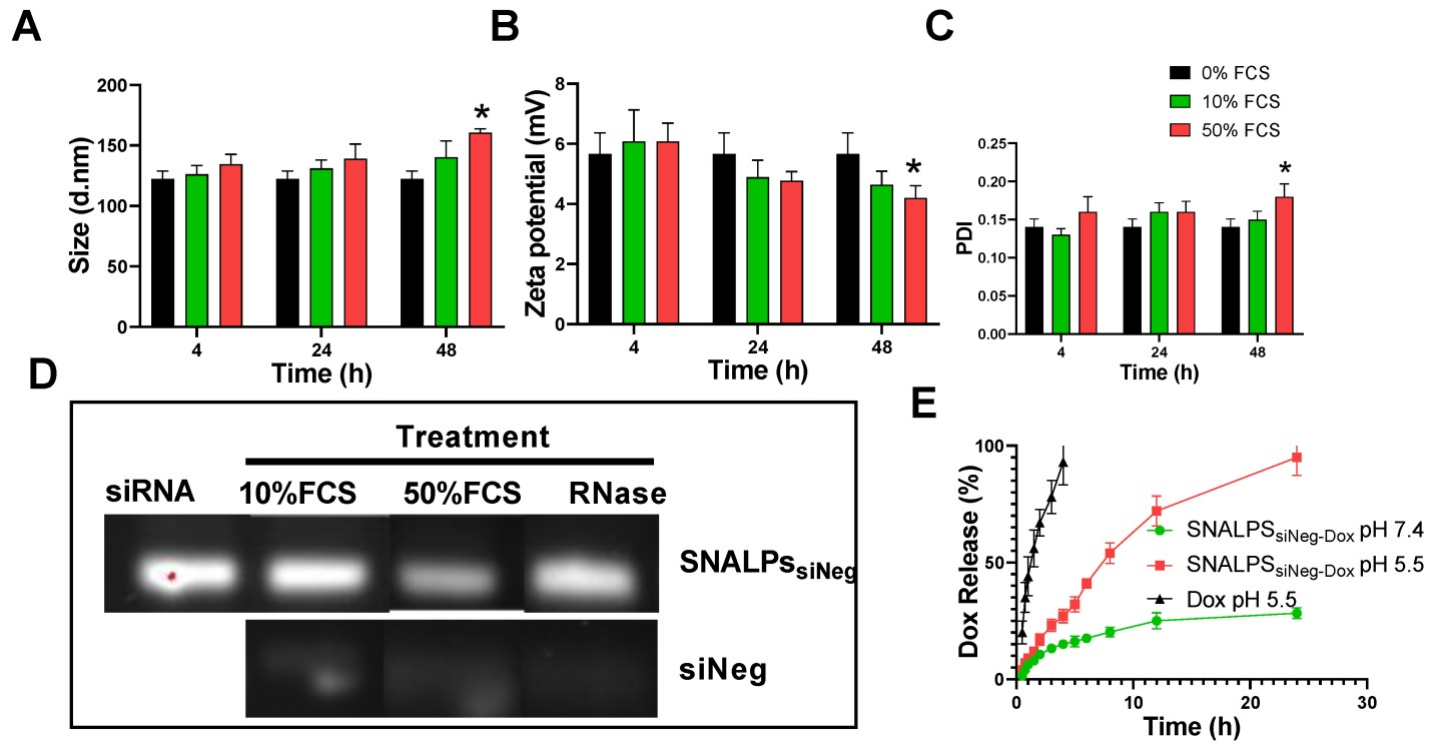
** Physicochemical characterization of SNALPs formulations.** SNALPs_siNeg_ were formulated as described before being incubated with either 0%, 10% or 50% FCS v/v in PBS. Following 24h or 48h incubation SNALPs size (**A**), surface charge **(B)** and poly dispersity index (PDI) **(C)** were measured using dynamic light scattering. To measure stability of RNA within formulations, SNALPs were incubated with 10% and 50% v/v FCS or RNase (100µg/mL) for 24h. RNAse were then inhibited by addition of EDTA before SNALPs were disassembled with heparin (100 IU). Released RNA was qualitatively assessed using gel electrophoresis with free siRNA used as a positive control (**D**). Drug release from SNALPs was measured by dialysing SNALPs_siNeg_ in the presence and absence of 50% FCS against PBS (pH 7.4), or in acetate buffer (pH 5.5). Drug concentration in the dialyzate was assessed by measuring the absorbance at 480 nm. As a control, an experiment was set up concurrently in which the same quantity of doxorubicin (Dox) was dialysed against acetate buffer for comparison (**E**). Statistical analysis was carried out using ANOVA test followed by Tukey HSD test *p<0.05. Data point represent mean and SD (n=3).

To assess the biological stability of SNALPs *in vitro* under simulated conditions, SNALPs were incubated in the presence of 10% and 50% v/v serum. As shown in **Figure 1**, no significant changes were observed in particle size, PDI or zeta potential following incubation with 10% v/v FCS at any time ≤ 48 h **(Figure 1A-C)**. In contrast, incubation with 50% serum for 48 h resulted in a marked increase in size and PDI, while decreasing zeta potential. In addition to this, as shown in **Figure 1D** and** S3B**, the SNALPs system was able to partially (50% FCS) or completely (10% FCS or RNAse) protect the encapsulated siRNA from degradation as demonstrated by the RNA band obtained following particle dissociation. The unformulated siRNA demonstrated extensive degradation as indicated by faded band in **Figure 1D** and** S3B**.

The morphological architecture of the proposed SNALPs_siNeg-Dox_ could be depicted from the transmission electron micrograph presented in **Figure S4A**. The particles appeared almost spherical in shape. The average measured mean particle size (200 particles) was 127 ±28 nm (**Figure S4B**) which is in agreement with the size obtained by DLS (**Table 1**).

The *in vitro* release of Dox from SNALPs_siNeg-Dox_ was studied up to 24 h in PBS (pH 7.4 and 50%v/v FCS) and acetate buffer pH 5.5 **(Figure 1E)**. Acetate buffer pH 5.5 was included to ensure complete dissolution of Dox. SNALPs_siNeg-Dox_ demonstrated a minimum release of Dox in PBS pH 7.4, with only ≈28% being released across the duration of the study. Complete release of Dox was observed when SNALPs were incubated in acetate buffer pH 5.5 demonstrating pH-dependent release. As a further measure of biocompatibility, the SNALPs system was shown to prevent Dox induced hemolysis (**Figure S5A**).

### Intracellular uptake of Dox, siRNA and* in vitro* cytotoxicity studies

**Figure 2:**
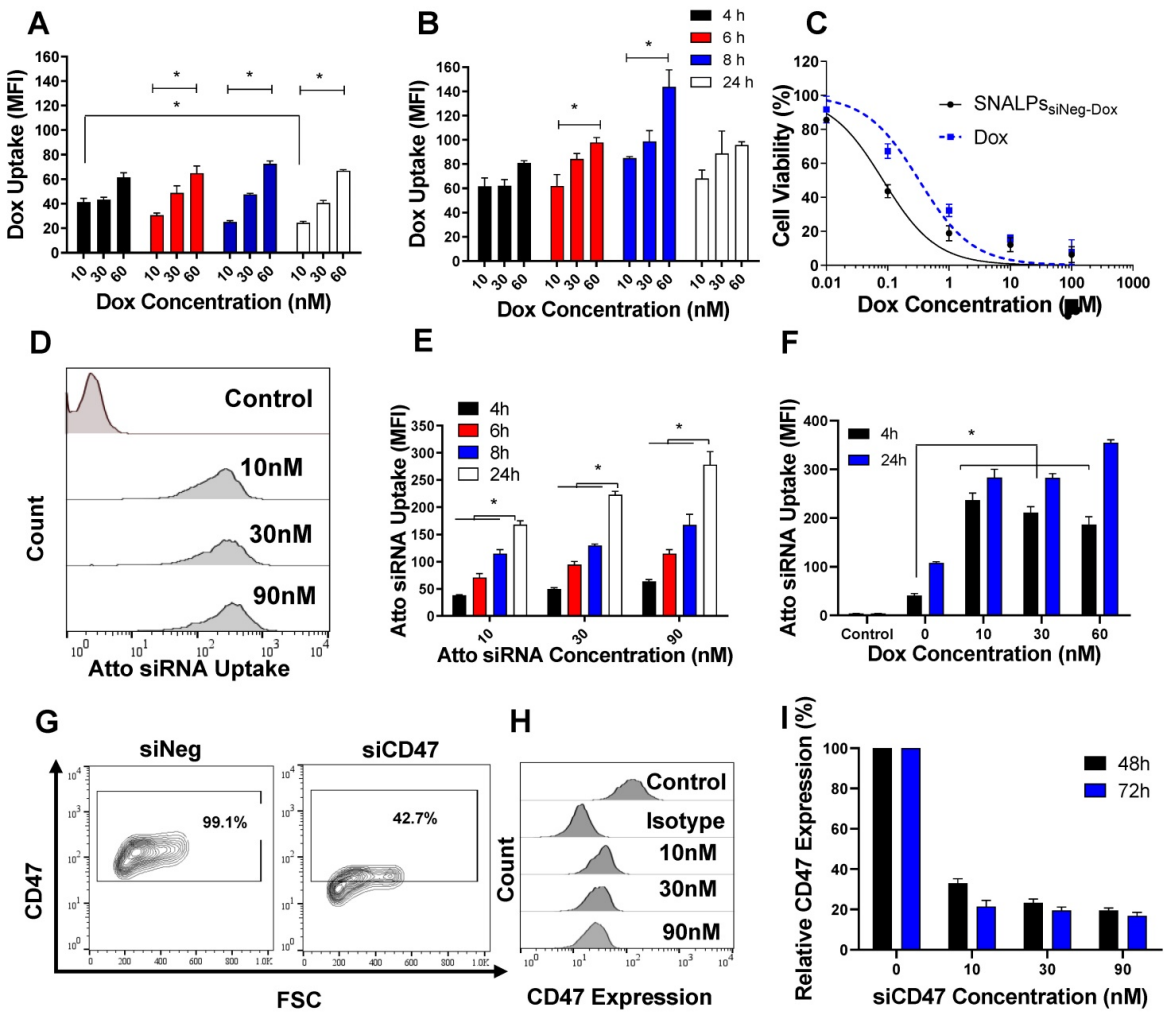
** Intracellular uptake, cytotoxicity and silencing efficiency of SNALPs in CT26 murine colon carcinoma cells *in vitro*.** CT26 cells were incubated with either the soluble Dox or SNALPs_siNeg-Dox_ at a range of concentrations for 4, 6, 8 and 24 h before being analyzed by flow cytometry. Cellular uptake across the entire study was assessed by measuring the mean fluorescence intensity (MFI) using flow cytometry (n=3) The uptake of Dox either free (**A**) of loaded into SNALPs (**B**) were assessed in CT26 cells over 4, 6, 8 and 24 h. SNALPs demonstrated increased Dox uptake compared to soluble Dox at all concentrations and time points tested. To measure cytotoxicity of the formulation, CT26 cells were incubated with either Dox or SNALPs_siNeg-Dox_ for 48 h at increasing drug concentrations (0.01- 100 μM). Cell viability was determined by MTT assay and data is presented as viable cells as a percentage of non-treated cells (n= 5) (**C**). Intracellular delivery of SNALPs_siAtto655_ at concentrations 10, 30, 90 nM after 4 and 24 h was assessed using flow cytometry. Representative flow cytometry histograms obtained at the 24 h time point are shown in (**D**). Quantitative uptake of siRNA expressed as MFI is shown in (**E**). siRNA uptake was higher at increasing concentrations and incubation times (* p<0.05). The effect of Dox on the uptake of SNALPs_siAtto655-Dox_ is shown in (**F**). Cells were incubated with SNALPs_siAtto655-Dox_ at increasing concentrations of soluble Dox (10, 30 and 60 nM) and fixed Atto 655-siRNA (30 nM). Uptake was assessed by flow cytometry as described. Co-incubation with soluble Dox increased uptake of siRNA in cells only at an earlier timepoint of 4h (*p<0.05). To evaluate gene silencing, CT26 cells were incubated with SNALPs_siCD47_ at three different siCD47 concentrations (0, 10, 30 and 90 nM) at 48 and 72 h. Representative flow cytometry contour plot for 30 nM siRNA at 48 h is shown in (**G**). Gates were drawn based on isotype controls. Histograms for each concentration of siRNA at 48 h is shown in (**H**). The knock-down efficiency of CD47 is presented as MFI as percentage untreated control which has been normalized to 100% (**I**). Data points represent mean and SD (n=3). Statistical analysis was performed using One-way ANOVA followed by Tukey's post-test *p<0.05.

Cellular uptake of SNALPs_siNeg-Dox_ was modelled in CT26 colon carcinoma by incubating cells with three different concentrations of the nano-formulation or soluble Dox (10, 30 and 60 nM Dox concentration) for 4, 6, 8 and 24 h. The intrinsic fluorescence of Dox, expressed as Mean Fluorescence Intensity (MFI), was used to quantify uptake. Representative flow cytometry histograms are shown in **Figure S5B**. **Figure 2A and B** illustrates a significantly higher Dox uptake in the form of SNALPs_siNeg-Dox_ over Dox solution at all concentrations and time points tested. Dox in the form of solution and SNALPs showed different uptake patterns. Dox solution showed maximum uptake at 4 h which was then reduced over time. On the contrary, SNALPs_siNeg-Dox_ showed a maximum uptake after 8 h. Expectedly, the improved uptake of SNALPs_siNeg-Dox_ resulted in higher *in vitro* toxicity compared to the soluble Dox at a dose range of 0.1-100 μM and 48 h incubation **(Figure 2C).** IC_50_ values of SNALPs_siNeg-Dox_ and Dox were calculated as 84 nM and 325 nM, respectively. The increased potency of SNALPs_siNeg-Dox_ at lower doses are likely due to improved uptake of Dox by the cells. **Figure S5C** confirmed that the enhanced toxicity for SNALPs_siNeg-Dox_ was not due to the cationic nature of the particles but due to higher Dox concentrations achieved in cells.

We then studied the uptake pattern of a fluorescently (Atto655)-labelled nonspecific siRNA (siAtto655) as a function of dose and time. Representative histograms are shown in **Figure 2D**. siAtto655 uptake in the form of SNALPs_siAtto655,_ in the absence of Dox, was time-and concentration-dependent (p < 0.05) **(Figure 2E)**. Co-incubation of cells with soluble Dox significantly increased the uptake of SNALPs_siAtto655_ compared to no Dox treatment at 4 h incubation while no Dox-induced enhancement in siRNA uptake was observed at 24 h **(Figure 2F)**.

### *In vitro* gene silencing

The ability of SNALPs_siCD47_ to knockdown CD47 expression in CT26 is illustrated in **Figure 2G-I**. Incubation of CT26 cells with SNALPs_siNeg_ minimally affected CD47 expression however a significant reduction in CD47 expression after treatment with SNALPs_siCD47_ was observed at all concentrations. Using the SNALPs based system developed, it was possible to reduce CD47 expression, as a percentage of control CD47 expression normalized to 100%, by 63% and 83% at 48 h and 72 h respectively with ≥ 30 nM siRNA. Increasing siCD47 concentration from 10 to 30 nM significantly increased the transfection efficiency from 67.03± 2.21 to 76.69± 1.89 (p < 0.05). On the contrary, incubation of CT26 cells with higher siCD47 concentration (90 nM) did not result in any increase in the gene silencing efficiency. Therefore, SNALPs_siCD47_ equivalent to 30 nM siCD47 was used as the maximum effective siRNA dose. The presence of Dox did not interfere with the silencing efficiency of SNALPs_siCD47-Dox_ **(Figure S6)**.

### SNALPs containing Dox can induce hallmarks of immunogenic cell death (ICD)

**Figure 3:**
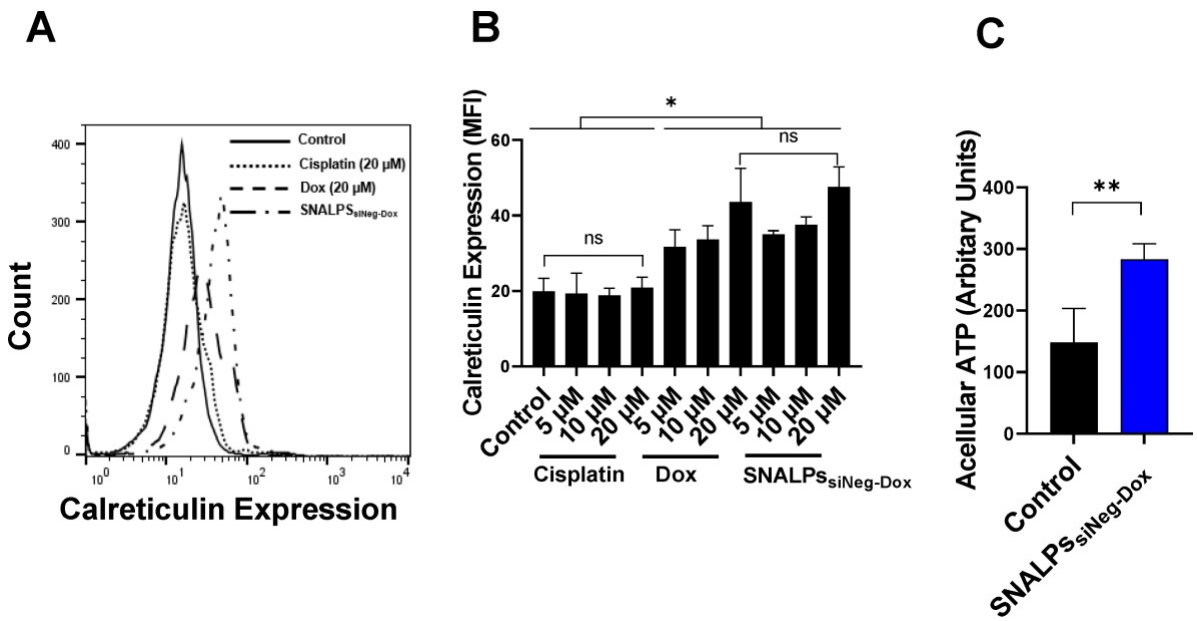
** Effect of Dox on calreticulin expression on CT26 murine colon cancer cells *in vitro*.** CT26 cells were pulsed with either cisplatin (5, 10 and 20 µM), Dox or SNALPs_siNeg-Dox_ (0, 5, 10 and 20 µM Dox) for 4 h before being stained with anti-calreticulin monoclonal antibody and acquired by FACs Calibur flow cytometer. Representative expression of calreticulin is shown in overlaid flow cytometry histograms (**A**). Cellular expression of calreticulin for each of the groups was calculated from the relative mean fluorescence intensity (MFI) (**B**).Statistical analysis was performed using ANOVA test followed by Tukey HSD test. Incubating CT26 cells with either Dox or SNALPs_siNeg-Dox_ significantly increased calreticulin expression when compared to either untreated cells or that incubated with the same concentrations of cisplatin (p< 0.05). To measure acellular ATP, CT26 cells were either left untreated as a control of pulsed with SNALPs_siNeg-Dox_. The cell supernatants were removed, and cellular debris cleared by centrifugation. ATP was quantified by luciferase-based ATP detection reagent (**C**). Statistical analysis was performed using Student T-test followed by Mann Whitney post-test *p<0.05, **p<0.005, ns non-significant. Bars represent mean and SD (n=3-6).

To demonstrate that SNALPs containing Dox can induce ICD two readouts were used: surface calreticulin expression and increased acellular ATP. In the first case, CT26 cells were exposed to either soluble Dox, SNALPs_siNeg-Dox_, cisplatin or left untreated. As shown in **Figure 3A**, cells treated with either Dox or SNALPs_siNeg-Dox_ displayed a significant increase in surface calreticulin over untreated control CT26 cells or those incubated with equi-concentrations of cisplatin (p < 0.05). This effect was shown to be concentration-dependent, ranging from 1.5-2.3-fold over basal levels **Figure 3B**. The formulation of Dox in a SNALPs system exhibited equivalent increases in expression when compared to soluble Dox. Cisplatin solution in the same concentration range, used as an example of a non-ICD inducing drug, did not induce calreticulin upregulation. In addition to surface calreticulin expression, the induction of ICD is associated with release of DAMPs such as acellular ATP. To test this CT26 were exposed to SNALPs_siNeg-Dox_ and ATP in cell supernatant was tested using luciferase-based assay. As shown in **Figure 3C**, exposure of cells to the formulation resulted in a significant increase in acellular ATP. The combined readouts strongly suggest that SNALPs containing Dox can induce ICD to equivalent levels as soluble Dox.

**Figure 4:**
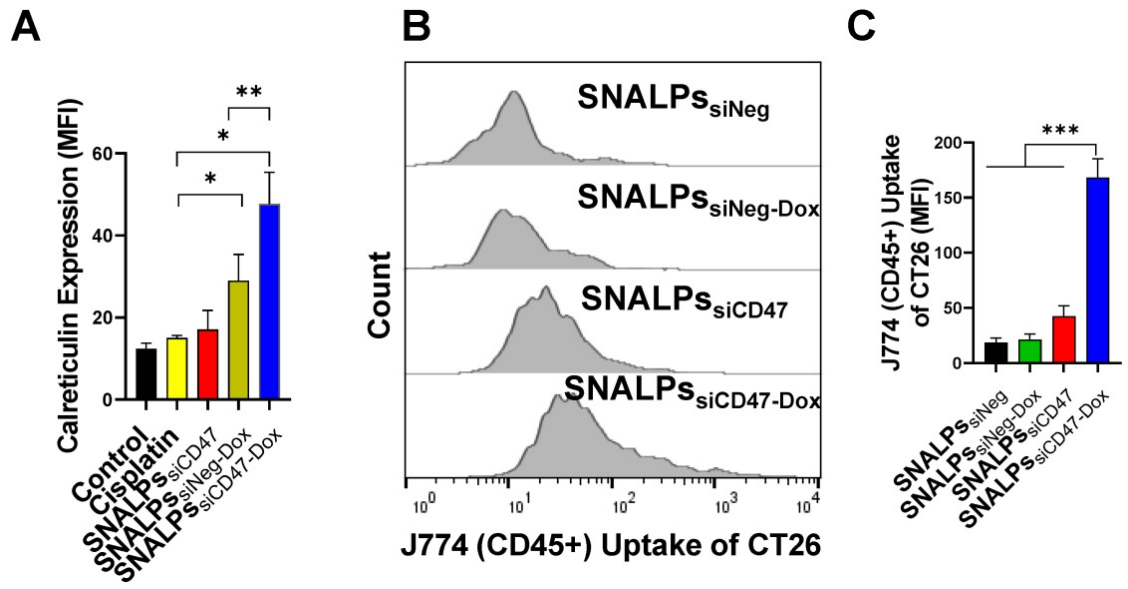
** Effect of incubation of CT26 murine colon cancer cells by different SNALPs on macrophage uptake.** The ability of both siCD47 and Dox containing SNALPs to alter expression of calretculin on cells was tested using flow cytometry. CT26 cells were pulsed with either PBS (control), cisplatin, SNALPs_siNeg-Dox_, SNALPs_siCD47_ or SNALPs_siCD47-Dox_ for 48 h (30 nM siRNA and 5 µM Dox/Cisplatin). Cells were harvested and stained with anti-calreticulin monoclonal antibody prior to acquisition on FACS Calibur flow cytometer^TM^. Data is expressed as mean fluorescence intensity and SD (n=3-6) (**A**). Statistical analysis was performed using a Student T-test followed by Mann Whitney post-test * p<0.05 **p<0.005. CT26 cells were labelled with CellTrace^TM^ before being incubated with SNALPs_siNeg_, SNALPs_siNeg-Dox_, SNALPs_siCD47_ and SNALPs_siCD47-Dox_ at concentration 30 nM and 60 nM for siRNA and Dox, respectively, for 48 h. The cells were collected and co-cultured with J774 macrophage cells for 6 h. Cells were harvested and stained with anti-mouse CD45 monoclonal before being acquired on a FACs Calibur flow cytometer. For analysis, cells were first gated on CD45 expression before CellTrace^TM^ fluorescence was quantified in CD45+ population, a representative histogram for each group is shown in (**B**). Relative MFI of CD45+ J774 cells is shown in (**C**). Bars represent the average of mean fluorescence intensity and SD (n=3). One-way ANOVA followed by Tukey's post-test ***p<0.001.

Interestingly, the SNALPs_siCD47-Dox_ at a concentration equivalent to 30 nM siCD47 and Dox 5 µM for 48 h was able to significantly elevate the calreticulin compared to SNALPs_siNeg-Dox_ or control untreated cells by ~2 or 4-fold respectively (**Figure 4A**). On the other hand, SNALPs_siCD47_ had insignificant effect on calreticulin expression compared to either untreated cells or incubated with cisplatin as an example of a non-ICD drug. The consequence of this on phagocytosis was investigated by macrophage uptake of target cells. Target cells were prepared by labeling CT26 with CellTrace before being treated with either SNALPs_siNeg_, SNALPs_siNeg-Dox_, SNALPs_siCD47_ or SNALPs_siCD47-Dox_ at a concentration of 30 nM for 48 h followed by 6 h incubation of treated cells with J774 macrophage cells. A representative histogram for target cell uptake by J774 is shown in **Figure 4B** which demonstrates the uptake is not uniform in its pattern. The data is represented graphically in **Figure 4C**. The respective uptake of CT26 by J774 (expressed as MFI) was 18.36± 4.42 and 42.63± 9.35 for siNeg (black bar) and siCD47 (red bar) treated groups which is greater than 2-fold difference. Different studies emphasize the potential improvement in phagocytosis following the inhibition of CD47 29-31. SNALPs_siCD47-Dox_ however significantly increased the J774 cellular uptake by 4 and 8-fold over SNALPs_siCD47_ and SNALPs_siNeg-Dox_, respectively (p < 0.05). Although it is likely the average of SNALPs_siCD47-Dox_ is exaggerated due to the non-uniform profile of uptake. These data suggest a potential synergistic effect between CD47 knockdown and Dox treatment.

### *In vivo* biodistribution

**Figure 5:**
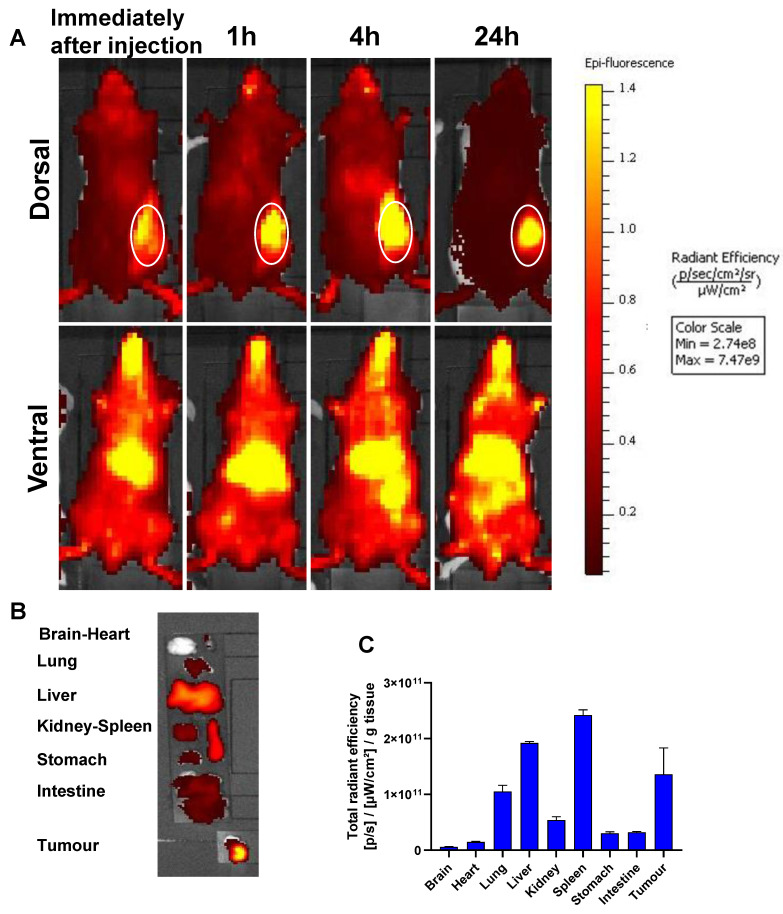
***In vivo* whole body IVIS imaging and biodistribution of DiR-labelled SNALPs_siNeg_ in CT26 tumour-bearing BALB/c mice after systemic administration.** Mice were inoculated subcutaneously with 1 x 10^6^ CT26 cells in right flank. When the tumour reached ~ 70-80 mm^3^, mice were i.v. injected with 200 µl of DiR labelled (1 mole%) of total lipid) SNALPs_siNeg_ in HEPES buffer pH 7 containing approximately 1 nmole siNeg. Animals were imaged using IVIS imaging system, (**A**) shows representative whole body images using 750/780 nm for DiR labelled SNALPs_siNeg_ tracking, immediately after injection and at 1, 4 and 24 h post-injection. The tumour site is highlighted. Animals were culled at 24 h post-injection and their organs were excised for analysis (**B**). *Ex vivo* organ biodistribution profile of DiR labelled SNALPs_siNeg_ in CT26 tumour-bearing BALB/c mice normalised to organ weight is shown in (**C**). Bars represent mean and SD (n=3).

To analyze the biodistribution and the tumor accumulation of the formulations, SNALPs_siNeg_ was prepared with the lipid label DiR. **Figure 5** shows the whole-body imaging of mice injected with a single i.v. injection over the course of 24 hours. The SNALPs displayed a relatively long circulation profile, with preferential liver and tumor accumulation in the early time points. At postmortem, organs were individually imaged and high quantities of SNALPs were detected in spleen and lung in addition to liver and tumor. The following trend was observed for SNALPs accumulation: Spleen > Liver > Tumor > Lung.

### Therapeutic efficacy of the prepared formulations

**Figure 6:**
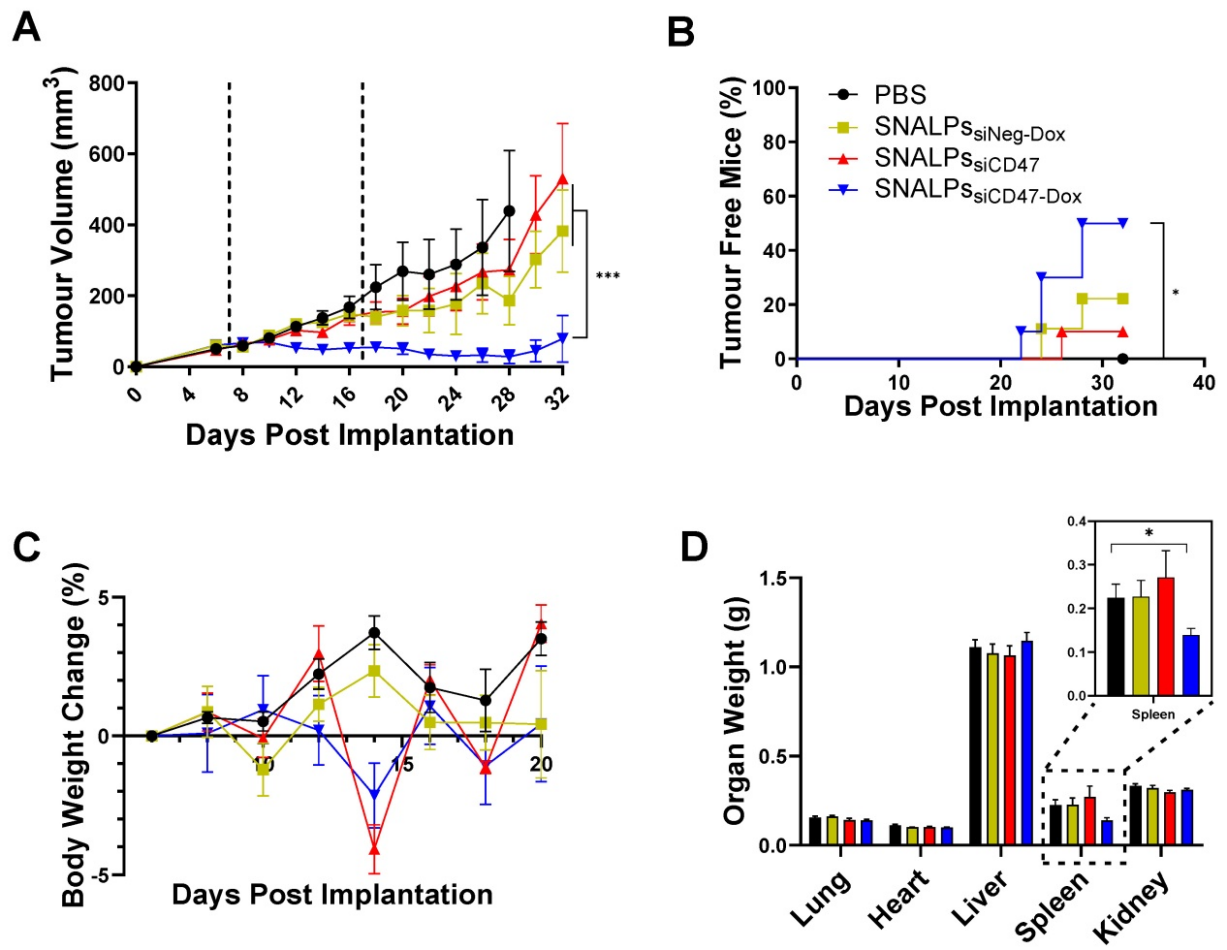
***In vivo* assessment of SNALPs in CT26 tumour model.** Mice (BALB/c n=10 per group) were implanted subcutaneously with 1 x 10^6^ CT26 cells. On day 7 and 17 (dashed lines), mice were i.v. injected with either PBS, SNALPs_siNeg-Dox_, SNALPs_siCD47_ or SNALPs_siCD47-Dox_. Dox was used at (5 mg/kg) while siRNA was used at (0.1 mg/kg). Tumour size was monitored for each mouse until the sacrifice of animals (**A**). Statistical analysis was performed using 2-way ANOVA followed by Tukey post-test, ***p<0.001, data points represents the mean and SEM. The percentage of mice resolving the tumour was recorded (**B**). Statistical analysis was carried out using a Mantel-Cox test *p<0.05. Changes in mouse weight relative to starting weight throughout the time course is reported in (**C**). At the terminal time point, mice were culled and individual organ weight was recorded, spleen weight has been magnified (inset) (**D**). Statistical analysis was performed using Student's T-test *p<0.05. Bars represent Mean and SEM.

To determine the therapeutic efficacy of the devised formulation, SNALPs_siCD47-Dox_ was compared with either SNALPs_siNeg-Dox_, SNALPs_siCD47_ or PBS treated animals in a CT26 tumor model. A two-dose regime was utilized with doses administered at days 7 and 17 **(Figure 6)**. The siRNA dose used in the *in vivo* study was selected based on previous reports 32, 33 suggesting siRNA dose of 0.1 mg/kg. The recommended therapeutic Dox dose for animal studies is 5 mg/kg 34. The concomitant use of siCD47 and Dox resulted in a significant tumor growth inhibition in comparison to other groups (p < 0.05) **(Figure 6A and S7)**. Furthermore, 50% of animals treated with SNALPs_siCD47-Dox_ no longer bore a palpable tumor at the end of the study compared to no spontaneous remissions in PBS treated mice **(Figure 6B)**. As a result of this, the study was terminated prior to humane endpoints to allow for the immunological assessment of remaining tumors. The tolerability of the prepared SNALPs was investigated by monitoring the change in body weight as well as the vital organ body weight at the terminal time point **(Figure 6C)**. None of the tested animals showed a loss of body weight ≥ 5% which indicated general biocompatibility of all the SNALPs formulations. Moreover, no significant difference could be detected by comparing the organ weight of the animals from groups with the exception of the spleen (p > 0.05) **(Figure 6D)**. Interestingly, the weight of the spleen in animals treated with SNALPs_siCD47-Dox_ was significantly less than that of other groups (p < 0.05).

No obvious pathological changes were observed in H&E stained vital organs (i.e. heart, lung, liver, spleen and kidney) in all treatment groups compared to PBS group **(Figure S8)**. The results suggest the therapeutic approach led by SNALPs delivery induced systemic and local activation of the immune system but did not exhibit general toxicity in mice.

### Immunohistological and immunological assessment of SNALPs formulation

**Figure 7:**
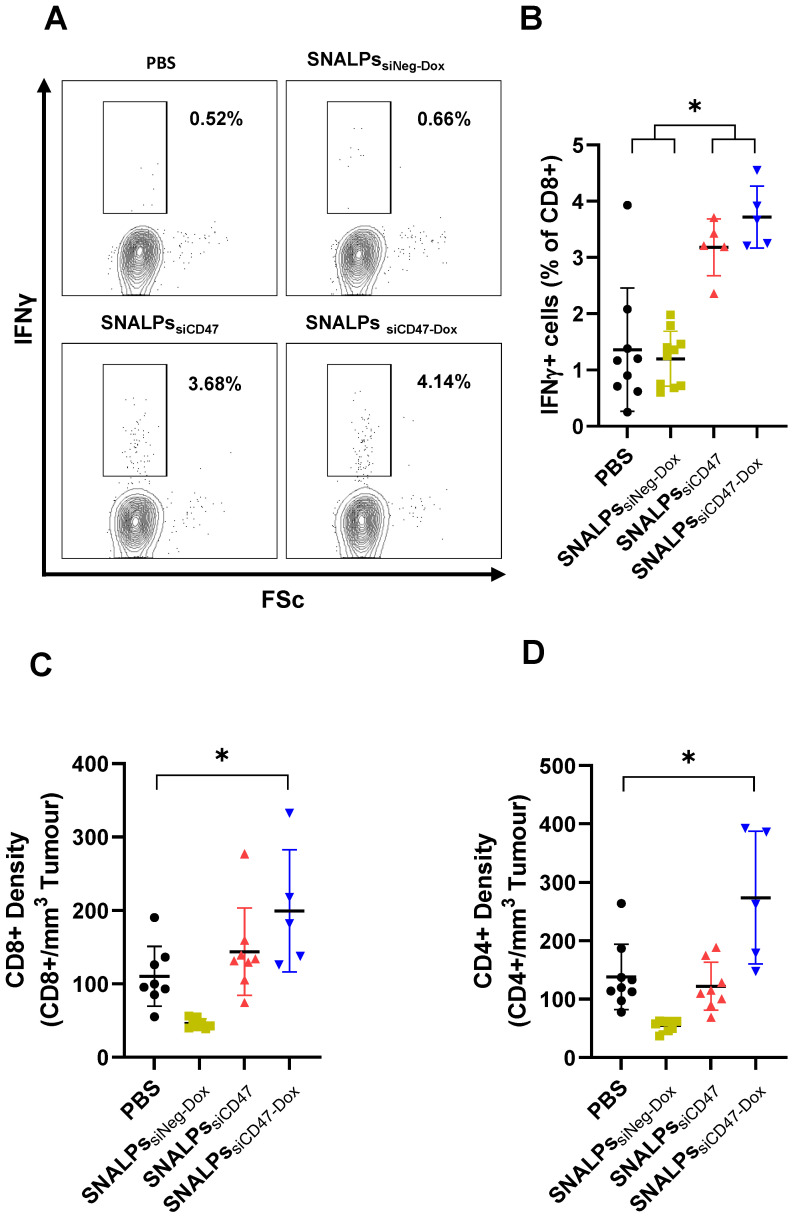
** Co-delivery of siCD47 and Dox in SNALPs significantly alters the immunological outcomes of tumour challenge.** Following termination of previously described experiment, mice were analysed for several immunological parameters. Cells were extracted from tissues by physical dissociation: tissues analysed included spleens (**A, B**) and tumours where present and (**C, D**). Splenocytes were cultured in the presence of brefeldin A for 6 h. Cell surface was stained with anti CD8 before being stained intracellularly with Anti IFNγ-APC. Cells were acquired on a FACs Calibur^TM^ flow cytometer and gated based on FSC/SSC. Representative flow plots CD8+ fraction population is shown in (**A**). The corresponding data is expressed graphically in (**B**). Cells extracted from tumour were stained with Anti CD8α-PE and CD4-FITC and acquired as described. Cells were first gated based on FSC/SSC profile before the relevant marker was assessed. Absolute cell numbers gathered from tumours were first normalised for tumour volume and presented as relative cells/mm3 for both CD8 (**C**) and CD4 (**D**). Data analysis was performed using Graphpad Prism *p<0.05 Student's T-test.

To measure immune responses, spleens and tumors were taken from mice on day 32 post-implantation. Systemic immune responses were measured by culturing splenocytes in the presence of brefeldin for 4 h before staining for IFNγ. Interestingly, groups receiving siCD47, either as SNALPs_siCD47_ or SNALPs_siCD47-Dox_, had approximately 3-4 times the amount of CD8+ IFNγ+ producing cells in the spleen when compared to groups receiving PBS or SNALPs_siNeg-Dox_ **(Figure 7A-B)**. While there were slightly more CD8+, IFNγ+ cells in the SNALPs_siCD47-Dox_ compared to SNALPs_siCD47_ this difference was not significant. A similar trend was observed when analyzing CD8+ cell numbers within the tumor **(Figure 7C)**, with both SNALPs_siCD47-Dox_ and SNALPs_siCD47_ having elevated total counts. However, only SNALPs_siCD47-Dox,_ not SNALPs_siCD47_, reached statistical significance when compared to PBS. CD4+ cells were likewise at a higher density in the tumors of SNALPs_siCD47-Dox_ treated mice which was not the case for SNALPs_siCD47_ **(Figure 7D)**. Though it should be noted the cell numbers were very low generally.

### IHC of CD3^+^ T cells and F4/80^+^ tumor-associated macrophages

The effects of doxorubicin treatment and/or CD47 silencing were further assessed by immunohistochemistry. CD3^+^ T cells showed no preference or localization within tumors across all groups tested; however, only one tumor tissue was available for the SNALPs_siCD47-Dox_ group as most of the tumors were cured after the treatment and the remaining tumors were very small **(Figure S9A)**. F4/80^+^ macrophages showed increased tumor infiltration in groups that received treatment, either siCD47 or doxorubicin or both. These observations were corroborated by the quantification of staining density. There was no difference in CD3 staining density between groups and a trend towards increased F4/80 staining density in the treatment groups compared to the untreated group, with a significant difference between the doxorubicin-treated and untreated groups (**Figure S9B**, p < 0.02, t-test).

Interestingly, although the excision of tumors was conducted on day 32, over a relative long period post the last SNALPs treatments on day 17, immunostaining of CD47 demonstrated that CD47 expression was reduced on tumors of SNALPs_siCD47_ group compared to PBS group **(Figure S10A)**. Necrosis was observed in the center of the only tumor in the SNALPs_siCD47-Dox_ group and low CD47 expression was detected. Quantitative analyses of the percentage of CD47 expression area (expressed as %DAB) and the staining intensity confirmed significant reduction of CD47 after SNALPs_siCD47_ therapy (**Figure S10B**, p = 0.02, t-test).

## Discussion

In this study, we developed stable nucleic acid-lipid particles (SNALPs) capable of encapsulating both siRNA and chemotherapeutic drug. Furthermore, we demonstrate potential synergy between ICD inducing drug and blockade of CD47. This synergy has previously been described to some extent with anti-CD47 monoclonal antibodies 35, 36. In this study, DOTAP is used as a cationic lipid to efficiently encapsulate siRNA. DOTAP is widely used in several nucleic acid loaded nanocarriers either *in vitro* or *in vivo* studies 37, 38 To load Dox, the clinically relevant pH remote loading technique was utilized 26, 39. The loading of Dox did not affect the encapsulation efficiency of siRNA. In contrast, the co-loading of siRNA and Dox significantly decreased the encapsulation efficiency of Dox. This could be explained by the sequential loading of siRNA followed by Dox, where siRNA could occupy some of the vacant space available for Dox loading. Expectedly, Dox demonstrated pH-dependent release profile due to drug protonation at acidic pH increasing its aqueous solubility 40.

The obtained SNALPs size, regardless of loading, fell within the desired range of between 100-200 nm, particles of this size have been shown to selectively accumulate within tumors due to the EPR effect 41, 42. Incubation with 50% serum for 48 h resulted in slight but significant increase in particle size, the concomitant increase in NPs size with adsorption of serum protein is widely reported in literature 43-46.

In the cellular uptake study, we decided to use a low concentration range of doxorubicin to maintain cell viability in order to investigate the effect of both concentration and time on the uptake. Since our hypothesis was to investigate the possible synergistic effect of combining siCD47 and ICD inducer drug, we utilized a low siRNA dose (less than 100 nM). Cellular uptake studies of Dox showed SNALPs improved intracellular uptake by almost 1.5-fold compared to Dox solution. Unlike SNALPs_siNeg-Dox_, free Dox showed reduced uptake by cells at later time points (> 4 h) possibly due to drug efflux, a phenomenon that has been widely reported in previous studies 47-49.

In accordance with improved uptake, the superior anticancer activity of SNALPs containing Dox was observed *in vitro*. The improved cellular uptake and consequently the improved cytotoxicity could possibly be attributed to the fact that Dox is a substrate for the p-glycoprotein (Pgp) efflux mechanism 50. Similar results were reported when Dox was encapsulated into different nanocarriers such as polymeric micelles 50. Although, the doxorubicin mechanism of action is intracellular, by DNA synthesis inhibition, there is some evidence for the interaction between doxorubicin and cell membrane 51. Doxorubicin is an amphiphilic drug with dihydroxyanthraquinone ring bound by a glycosidic bond to the amino sugar. The dihydroanthraquinone residue is responsible for the hydrophobic interaction with the lipid portion of the cell membrane 52. Moreover, the amino sugar is responsible for the molecule's positive charge which in turn facilitates the electrostatic interaction between doxorubicin and cell membrane. This interaction is believed to affect the cell membrane integrity and decreased the membrane microviscosity 52. Since the cell membrane is the main barrier for cellular internalization of siRNA, as a polyanionic hydrophilic macromolecule, doxorubicin might improve the cellular internalization of siRNA. SNALPs containing Dox were shown to be able to upregulate surface calreticulin on CT26 to levels comparable to soluble Dox and were also able to cause the release of ATP. This suggests that the SNALPs system does not interfere with ICD. This has been reported for other particulate systems with ICD inducers 27.

In this study, SNALPs were successfully prepared and characterized for use as combinatory immunotherapy/chemotherapy agents containing both siCD47 and Dox. Under normal physiological conditions, there is a homeostatic balance between the 'eat me' signal, calreticulin, and 'don't eat me' signal CD47 53. Cancer cells have disturbed this axis by over expressing CD47 signals 14. Cancer cells develop this mechanism to evade clearance by the mononuclear phagocyte system 17. Liposomal and polyplex based systems have previously been used to deliver siCD47 in isolation or with other siRNA constructs (siPD-L1) 17, 53, 54. In these previous studies, the use of siCD47 resulted in significant inhibition of tumor growth in different models. Indeed, 6 doses of nanoparticles containing siCD47 resulted in near complete inhibition of tumor growth, an effect attributed to the activity of macrophages 17. It was speculated that the combination of ICD inducer and siCD47 would produce a synergistic effect on cell uptake. To measure this, surface calreticulin was stained and a macrophage cancer cell co-culture assay was utilized. The siCD47 monotreatment did not significantly increase calreticulin surface levels where both the Dox and the siCD47 plus Dox formulations did so. The difference in calreticulin expression between Dox and Dox plus siCD47 was an unexpected observation and has not been previously reported. It may represent mechanistic interplay between the two molecules or an artifact of our system. However, consistent with the calreticulin data, the co-culture of cancer cells treated with Dox and siCD47 showed a pronounced increase in uptake compared to either siCD47 or Dox.

It was shown that labelled SNALPs had a preferential accumulation into the subcutaneous tumor after intravenous injection, as the SNALPs featured no targeting moiety this is believed to be due to the passive EPR effect 55. Furthermore, as expected, the SNALPs were also accumulated in both liver and spleen as they are a part of the mononuclear phagocytic system as previously reported 22, 56-58. Following tumor challenge, it was revealed that the combination of siCD47 and Dox is extremely potent resulting in remission of 50% of tumors after two doses, while the remainder showed a significantly lower tumor volume compared with individual treatment alone or the PBS treated group. This is more potent than any of the previously reported studies utilizing siCD47 17, 53, 54. The minimal change in body weight within the different groups infers the biocompatibility of the prepared SNALPs as has been reported 21. We observed the spleens in mice of the SNALPs_siCD47-Dox_ were significantly smaller than the other groups, it has been reported that increased spleen weight can be associated with tumor progression in some models 59, though not typically for CT26. This remains an unexplained observation. In terms of measurable immune outcomes: it was observed that there were more CD8+ and CD4+ cells present in the tumors of the mice receiving combination Dox siCD47 SNALPs which could be responsible for reduced size. However, it should be noted that this data represents 50% of the group only, those mice which had not successfully cleared the tumor. This may not have been present in the mice resolving the tumor. In addition to local responses, there were high numbers of IFNγ secreting CD8+ cells present in the spleens of the siCD47 containing SNALPs. This could indicate the establishment of systemic immune responses or, as noted previously, a response to SNALPs accumulation within the spleen. This is in accordance with previous data where it was shown that siCD47 is able to activate the innate immune system 54. Though we have identified several immunological parameters which may contribute to the therapeutic efficacy, it should be highlighted this is observational data only and by no means represent a comprehensive assessment. From the data analyzed we cannot conclude the exact *in vivo* immunological mechanism. CT26 is known to be an 'immunogenic' cancer cell line and has been used in many of the early ICD experiments, whether these results are representative generally has yet to be determined 60.

## Conclusions

In conclusion, these data suggest that combining siCD47 with an ICD inducing drug (Dox) in a single formulation results in potent synergy in anti-tumor therapy and potentially could resolve certain types of 'immunogenic' solid tumors. There is data to suggest that this effect is likely to be due to a combination between the cytotoxicity of Dox, the increased infiltration of CD8+ cells and the systemic activation of the immune system possible as a result of ICD.

## Supplementary Material

Supplementary figures and tables.

## Figures and Tables

**Table 1:** Characterisation of selected SNALPs formulation

Formulation	siRNA	Dox	Size (d.nm)^a,d^	PDI^a,d^	Charge (mV)^a,d^	siRNA EE (%)^ b,d^	Dox EE (%)^ c,d^
SNALPs_siNeg_	+	-	114.33±6.51	0.17±0.012	5.23±0.42	61.29±4.45	-------
SNALPs_Dox_	-	+	107.66±3.21	0.19±0.006	6.35±0.56	-------	87.64±1.45
SNALPs_siNeg-Dox_	+	+	122.33±6.65	0.14±0.011	5.66±0.71	65.11±6.25	69.23±1.15

^a^ Measured by dynamic light scattering; ^b^ Calculated as percentage of initial siRNA added, determined by gel red method; ^c^ Calculated as percentage of initial doxorubicin added, determined by spectrophotometry; ^d^ Expressed as mean ± SD (n=3).
