# Supplementary material for: An “eat me” combinatory nano-formulation for systemic immunotherapy of solid tumors
Source: Theranostics. 2021 Aug 11;11(18):8738–54. doi: 10.7150/thno.56936 (PMC8419059; doi:10.7150/thno.56936)
Supplement: Supplementary file 1 — Supplementary figures and tables. [file thnov11p8738s1.pdf]

Supplementary Information

An "Eat me" Combinatory Nano-formulation for Systemic Immunotherapy of Solid Tumours

*Hend Mohamed Abdel-Bar[a, b‡], Adam A Walters[a‡], Yau Lim[a], Nadia Rouatbi[a], Yue Qin[a], Fatemeh Gheidari[a], Shunping Han[a], Rihab Osman[c], Julie Tzu-Wen Wang[a], Khuloud T. Al-Jamal[a, *]*

[a] Institute of Pharmaceutical Science, Faculty of Life Sciences & Medicine, King's College London, Franklin-Wilkins Building, 150 Stamford Street, London SE1 9NH, United Kingdom

[b] Department of Pharmaceutics, Faculty of Pharmacy, University of Sadat City, P.O. box: 32958 Egypt

[c] Faculty of Pharmacy-Ain Shams University, Abbassia, Cairo, P.O. box: 11566 Egypt

‡Contributed equally to the work

Corresponding author @:

Prof. Khuloud T. Al-Jamal

Email address: khuloud.al-jamal@kcl.ac.uk (K.T. Al-Jamal).

Table S1: Physicochemical characterisation of SNALPs formulations prepared throughout study

Payload	Formula code	Lipid composition molar%				Particle size (nm)[a, d]	PDI[a, d]	Zeta potential (mV)[a, d,]	siRNA EE(%)[b, d]	Doxorubicin EE (%)[c, d]
		Ch	DSPC	DOTAP	C16-Ceramide PEG2000					
siRNA	F1	45	22	25	8	130.66±6.02	0.11±0.008	9.06±1.13	50.25±3.24	-------
	F2	40	25	25	10	135±7	0.21±0.008	8.26±0.69	56.41±5.61	-------
	F3	45	25	20	10	114.33±6.51	0.17±0.012	5.23±0.42	61.29±4.45	-------
	F4	45	25	25	5	141.33±8.08	0.2±0.015	7.81±0.69	63.11±6.11	-------
Doxorubicin	F1	45	22	25	8	113±2.64	0.19±0.011	11.13± 1.11	-------	91.66±1.36
	F2	40	25	25	10	119±5	0.17±0.024	8.52±1.21	-------	80.65±1.59
	F3	45	25	20	10	107.66±3.21	0.19±0.006	6.35±0.56	-------	87.64±1.45
	F4	45	25	25	5	134.33±6.02	0.18±0.014	7.63±0.54	-------	95.35±0.79
Doxorubicin-siRNA	F1	45	22	25	8	136.66±5.51	0.15±0.021	8.39±1.26	47.11±3.98	75.07±1.4
	F2	40	25	25	10	142.33±7.23	0.19±0.021	6.39±1.22	59.3±4.93	63.71±1.26
	F3	45	25	20	10	122.33±6.65	0.14±0.011	5.66±0.71	65.11±6.25	69.23±1.15
	F4	45	25	25	5	158.33±5.51	0.17±0.008	6.54±0.79	60.12±3.91	71.34±2.17

[a] Measured by dynamic light scattering.
[b] Calculated as percentage of initial siRNA added, determined by gel red method.
[c] Calculated as percentage of initial doxorubicin added, determined spectrophotometry.
[d] Expressed as mean ± SD (n=3).

Table S2: Composition of the selected SNALPs formulation throughout different studies

Study	Initial composition										Final composition[a]			
	Total lipid		N/P ratio	Volume (µl)	siRNA		DiR		Dox		siRNA		Dox	
	Amount (µmole)	Concentration (mM)			Amount (nmole)	Concentration (µM)	Amount (nmole)	Concentration (µM)	Amount (nmole)	Concentration (µM)	Amount (nmole)	Concentration (µM)	Amount (nmole)	Concentration (µM)
In vitro gene silencing	0.213	2.13	4:1	100	1	10	---	---	75.82	758.27	0.65	6.5	52.32	523.21
In vivo imaging and biodistribution [a]	0.426	2.13	4:1	200	2	10	4.33	21.69	---	---	1.3	6.5	---	---
In vivo therapy study[b]	0.7	3.5	48:1	100	0.231	1.15	---	---	249.87	1249.37	0.15	0.75	172.41	862.06

[a] Lipid, siRNA and DiR dose is 21.99 mg/Kg, 0.8645 mg/kg and 0.22 mg/Kg, respectively.
[b] Lipid, siRNA and Dox dose is 36.23 mg/Kg, 0.1 mg/Kg and 5 mg/Kg, respectively.
[c] Lipid and DiR recovery was estimated 100%. siRNA, and Dox encapsulation efficacy was 65.11±6.25 and 69.23±1.15 respectively.

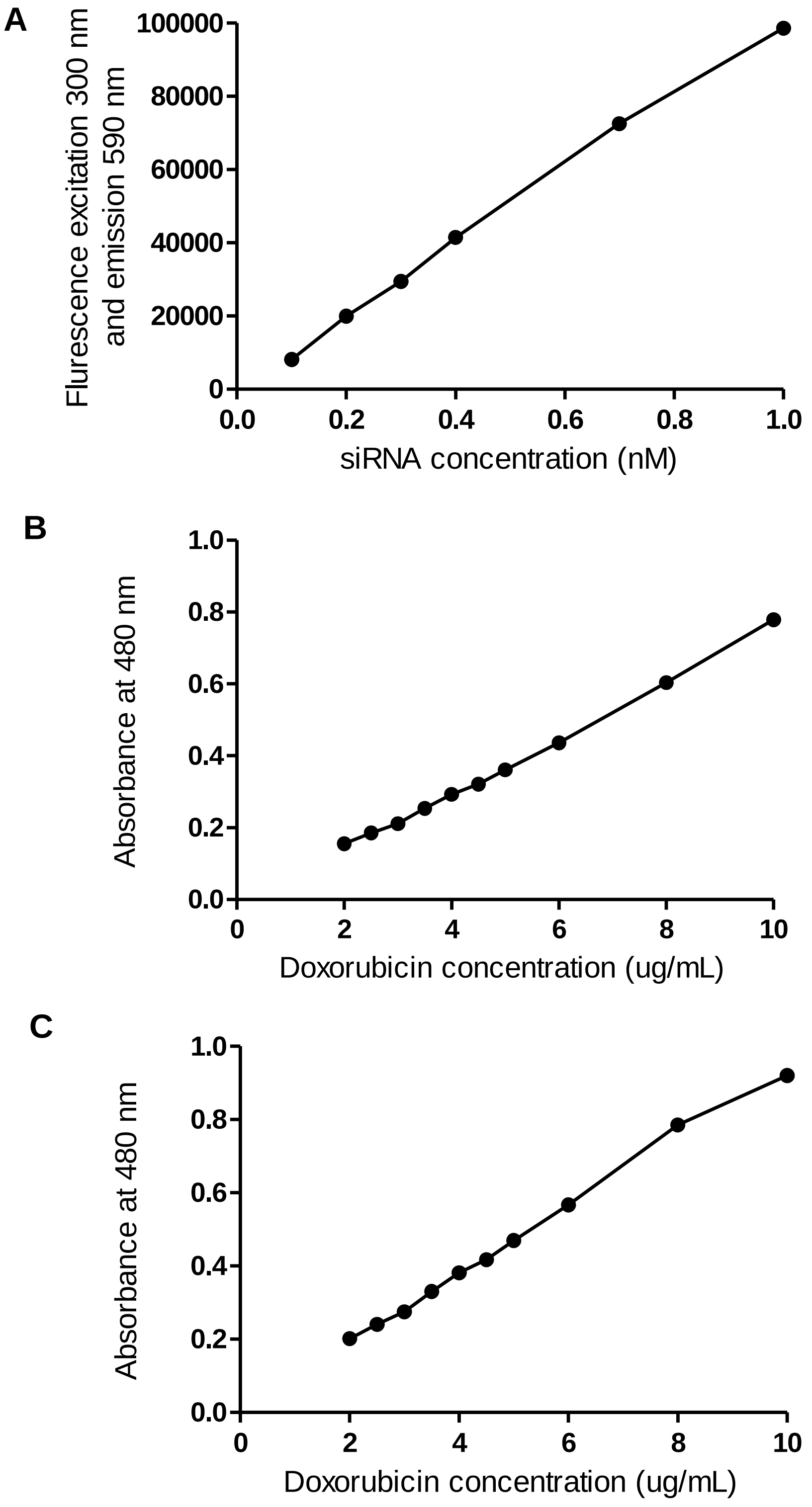

Figure S1. Standard Curves. (A) Calibration curve of siRNA in Tris buffer pH 7. siRNA was detected by Gel Red assay in the range of 0.2-1 nM by spectrofluorimetry at excitation and emission wavelengths of 300 and 590 nm respectively. Coefficient of variation was 0.9977. **(B)** Calibration curve of doxorubicin in PBS (pH 7.4) and **(C)** acetate buffer (pH 5.5) in the range of 2-10 ug/mL. Doxorubicin was detected by spectrophotometry at 480 nm absorbance. Coefficients of variation were 0.9968 and 0.9965 respectively.

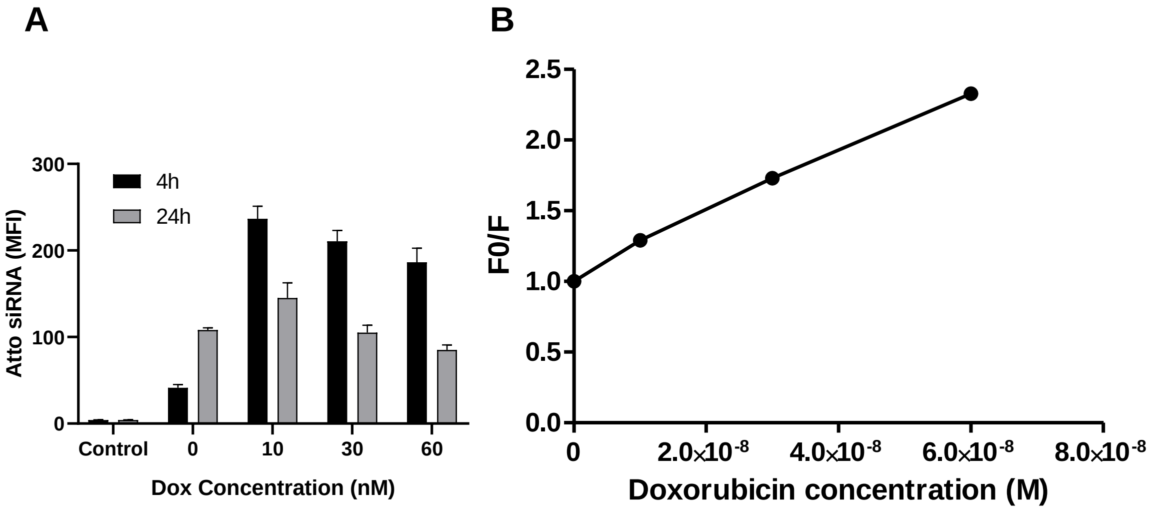

C

Dox concentration	OD at 655 nm	OD at 680	Correction factor
10 nM	0.31	0.285	1.95
30 nM	0.451	0.412	2.69
60 nM	0.61	0.634	4.17

Figure S2. Intracellular uptake of SNALPs$_{siAtto655}$ at 30 nM siRNA concentration in the presence of Dox at concentrations 10, 30 and 60nM after 4 and 24 h was assessed using flow cytometry. Quantitative uptake of siRNA, expressed as MFI is shown in (**A**). The apparent reduction in cellular siAtto655 uptake is due to the quenching effect of Dox. The Stern-Volmer plot of the Atto655-siRNA (30 nM) relative emission intensities as a function of the Dox concentration **(B)**. The correction of Dox quenching effect on siAtto655 florescence intensity **(C)**. The OD of different SNALPs$_{siAtto655}$ was determined at 655 nm and 680 nm. All measurements are the mean of three replicates. The quenched siAtto655 florescence intensity was corrected using the following equation:

$$F_{corrcted} = F_{observed}\, Log^{-1}\left(\frac{ODexcitation + ODemission}{2}\right)$$

A

B

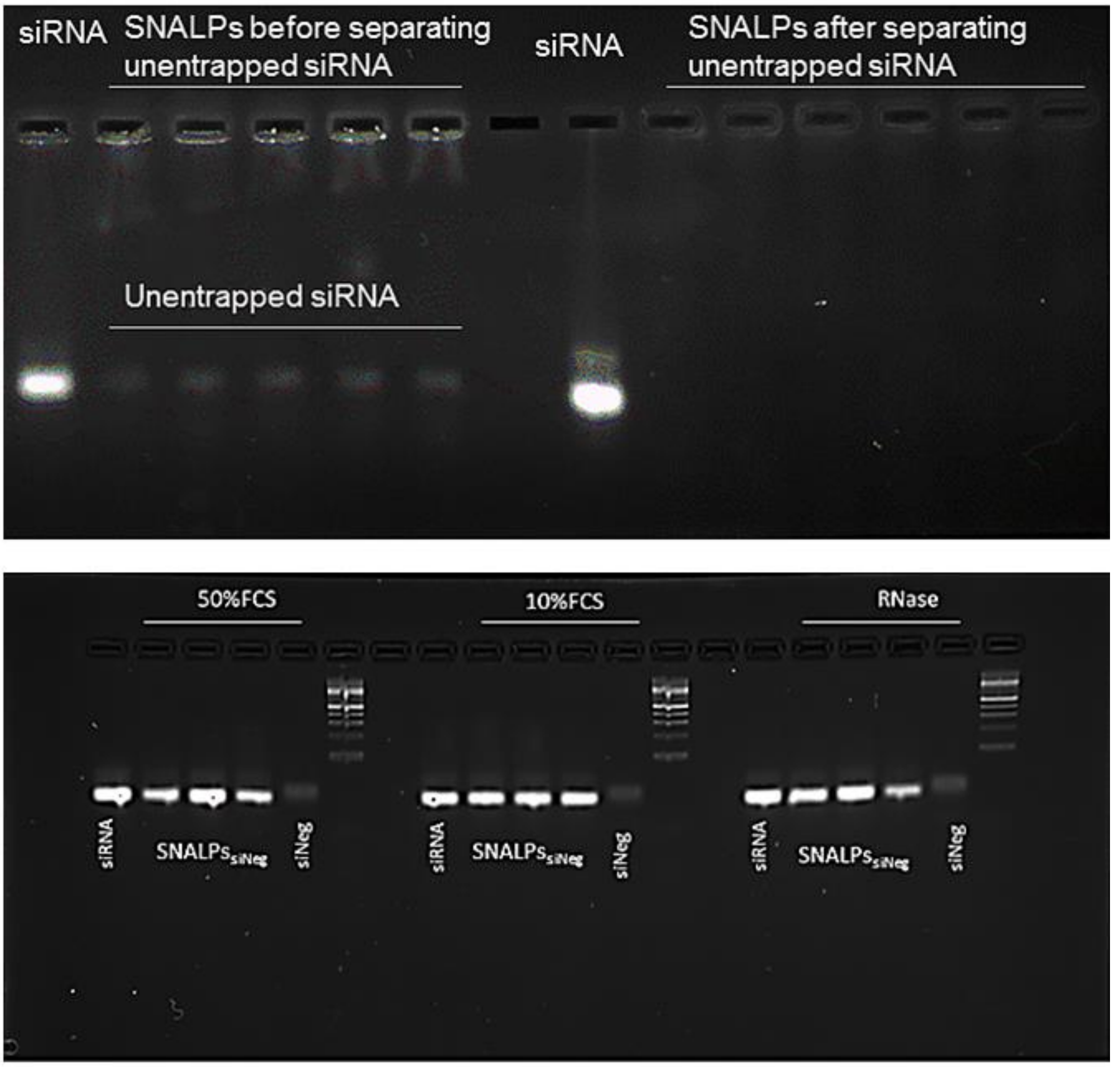

Figure S3. Gel retardation assay of SNALPs. The loading ability of siRNA into SNALPs$_{siNeg}$ **(A)**. SNALPs$_{siNeg}$ were formulated as described before. The loading was qualitatively determined by gel electrophoresis before and after removing unentrapped siRNA. To measure stability of siRNA within formulations SNALPs were incubated with 0% 10% and 50% v/v FCS in PBS or RNase (100µg/mL) for 24h **(B)**. RNAse were then inhibited by addition of EDTA before SNALPs were disassembled with heparin (100 IU). Released RNA was qualitatively assessed using gel electrophoresis with free siRNA used as a positive control. SNALPs were able to completely or partially protect the encapsulated siRNA from degradation in 10%, 50 % v/v FCS or RNase respectively. On the contrary, naked siRNA was extensively degraded in the assigned conditions.

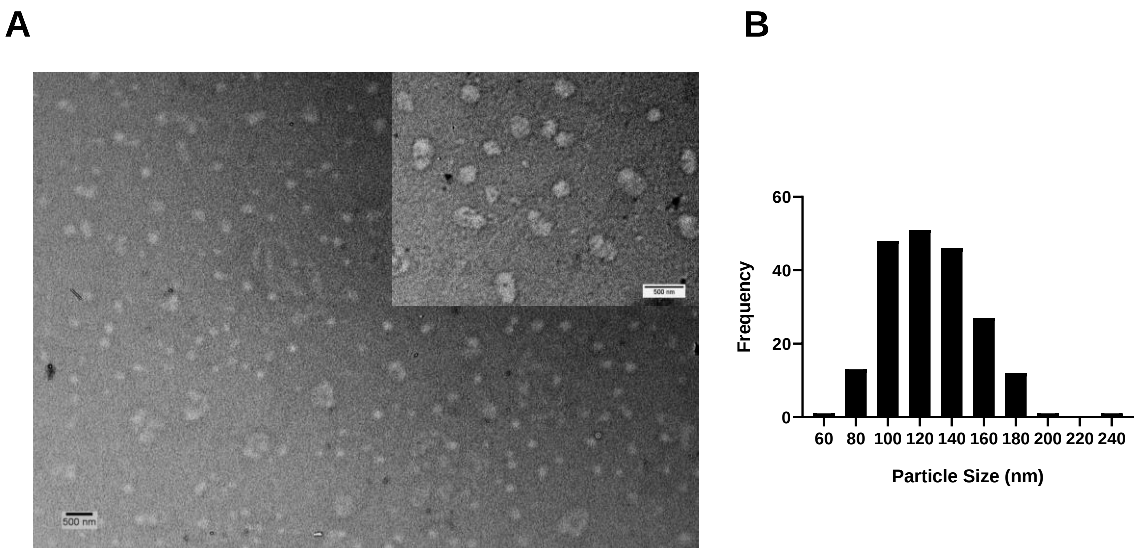

Figure S4. Morphological characterization of the proposed SNALPs. Transmission electron micrograph of $SNALPs_{siNeg\text{-}Dox}$ (scale bars = 500 nm) **(A)**. $SNALPs_{siNeg\text{-}Dox}$ appeared almost spherical in structure. Histogram of the SNALPs' size distribution where two hundreds particles have been counted with average particle size of 127 ± 28 nm (**B**).

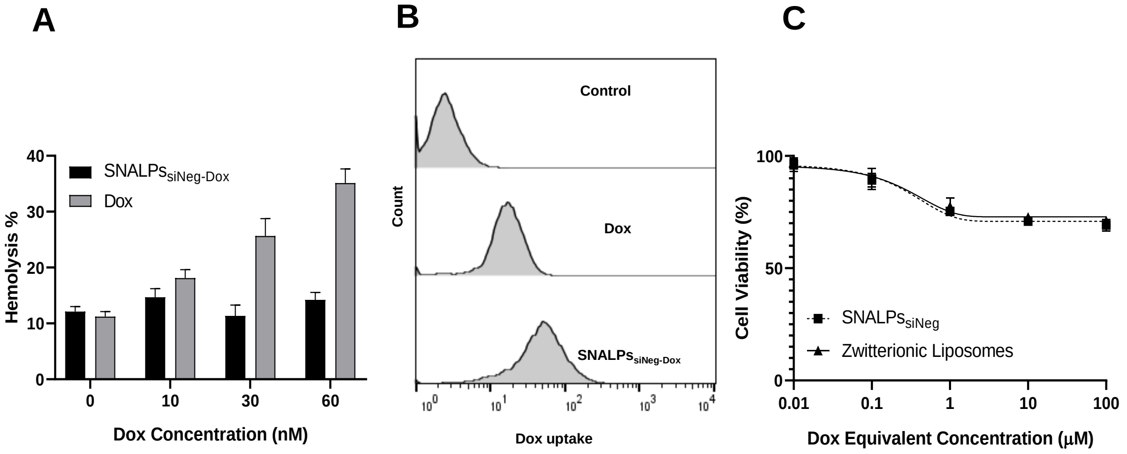

Figure S5. **Biocompatibility of SNALPs formulations and intracellular uptake of Dox solution and SNALPs. (A)** BALB/c erythrocytes were mixed with either free Dox or SNALPs$_{\text{siNeg-Dox}}$ at 0, 10, 30 or 60nM concentrations in PBS pH 7.4 for 2 h. Erythrocytes were also incubated with 0.5% Triton X as a positive control. Samples were centrifuged and supernatant absorbance at 540nm was determined. Haemolysis was presented as a percentage of haemolysis induced by Triton X-100^{TM} (100%). Bars represent mean and SD (n=3). (**B**) A representative flow cytometry histogram for uptake of 10 nM Dox or SNALPs$_{\text{siNeg}}$-Dox at 24 h is shown in. **(C)** To determine toxicity of lipid formulation, the cationic SNALPs were compared to zwitterionic liposomes. For comparative purposes, lipid concentration was equated to the corresponding lipid concentrations of SNALPs$_{\text{siNeg-Dox}}$ described in Fig 2. Cells were incubated with formulations for 48 h and cell viability was determined by MTT assay. Data is presented as viable cells as a percentage of non-treated cells. Mean and SD are plotted (n= 3).

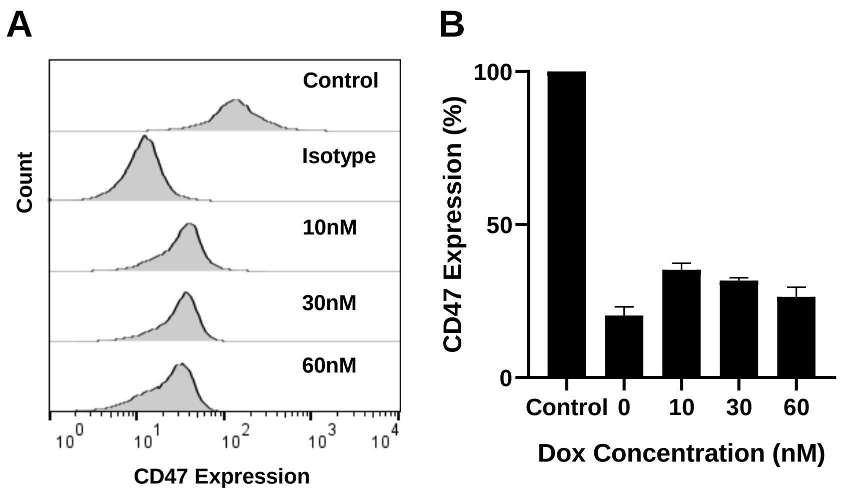

Figure S6. **CD47 knock-down is unaffected by the presence of Dox.** CT26 were treated with SNALPs$_{siCD47}$ (30nM siRNA concentration, 48 h) in the presence of soluble Dox at 0, 10, 30 or 60 nM. A group was left untreated as a control. Cells were harvested and stained with anti CD47 monoclonal antibody before being acquired on a FAC Calibur flow cytometer, a representative histogram is shown in (**A**). Expression was quantified using MFI and is presented as percentage CD47 expression compared to control normalised to 100% (**B**). Data is presented as mean and SD (n=3).

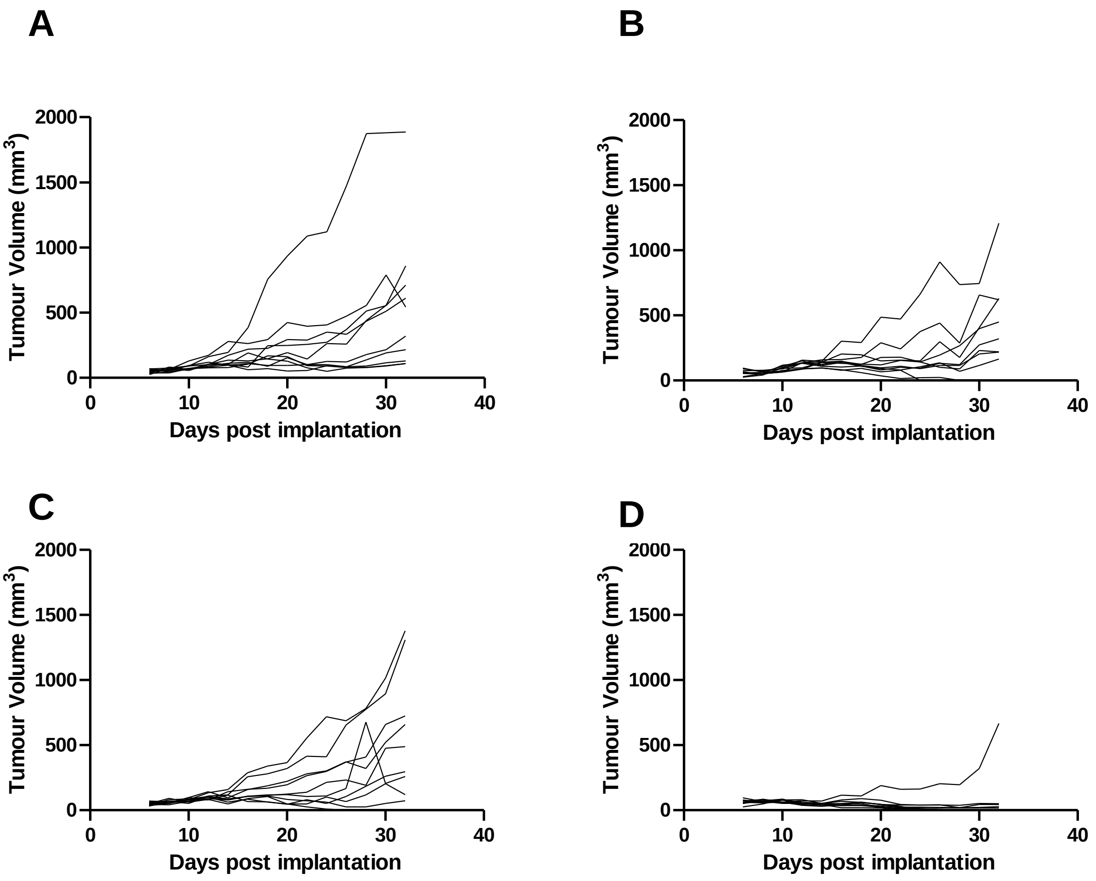

Figure S7. **Tumour volume measurements in CT26 tumour model.** Mice (BALB/c n=10 per group) were implanted subcutaneously with 1 x 10^6 CT26 cells. On day 7 and 17 mice were i.v. injected with either PBS, SNALPs$_{siNeg\text{-}Dox}$, SNALPs$_{siCD47}$ or SNALPs$_{siCD47\text{-}Dox}$. Dox was used at (5 mg/kg) while siRNA was used at (0.1 mg/kg). Tumour size was monitored every 2 days and represented for each mice in PBS (**A**), SNALPs$_{siNeg\text{-}Dox}$ (**B**), SNALPs$_{siCD47}$ (**C**) or SNALPs$_{siCD47\text{-}Dox}$ (**D**).

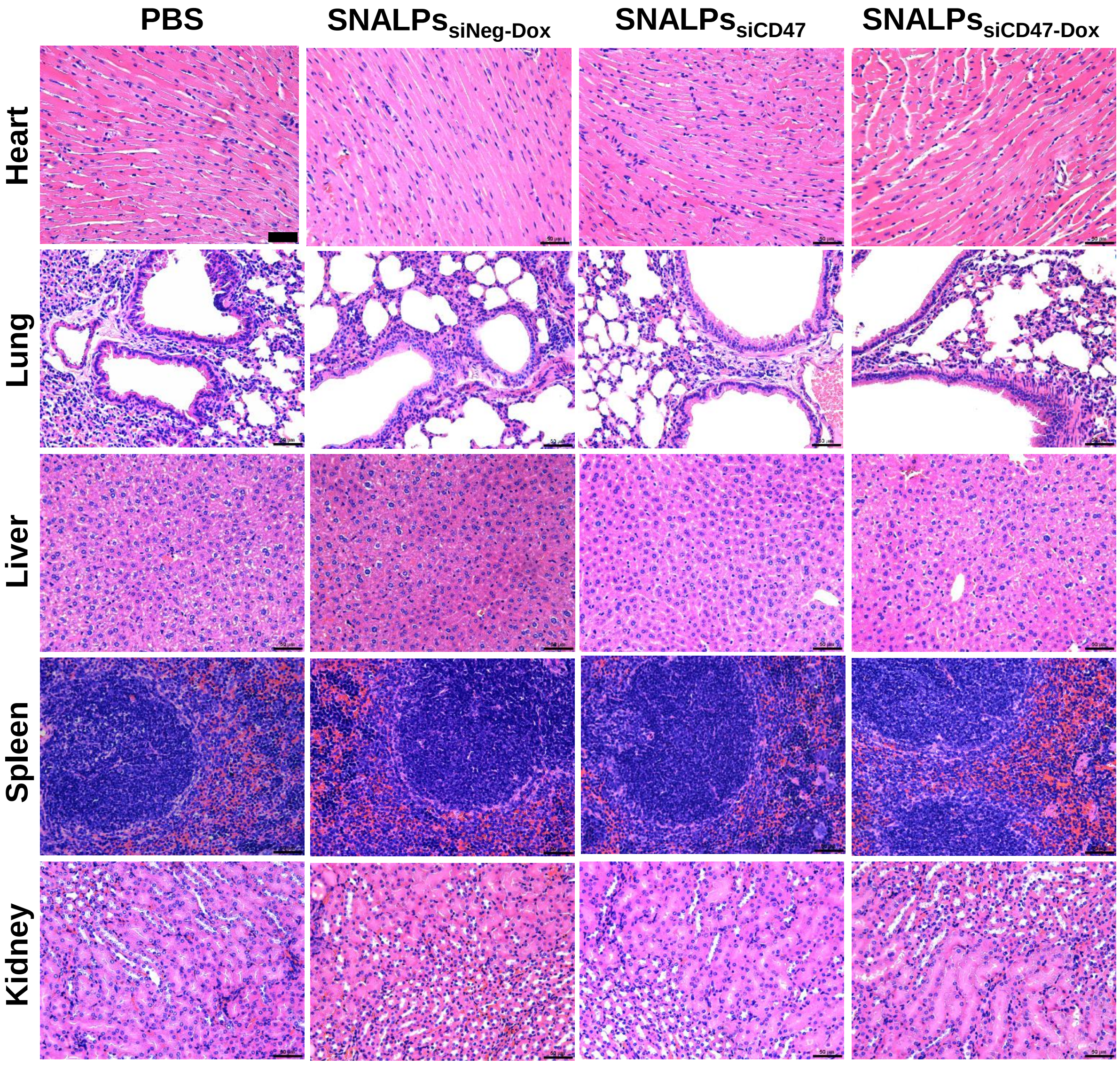

Figure S8. Histological examination of major organs after therapy studies. BALB/c mice were implanted subcutaneously with 1 x 10^6 CT26 cells. On day 7 and 17, mice were i.v. injected with either PBS, SNALPs$_{siNeg\text{-}Dox}$, SNALPs$_{siCD47}$ or SNALPs$_{siCD47\text{-}Dox}$. Dox was used at (5 mg/kg) while siRNA was used at (100 µg/kg). On day 32 the experimental end point, major organs (heart, lung, liver, spleen and kidney) were excised, formalin-fixed, and stained with H&E. Scale bar, 50 µm.

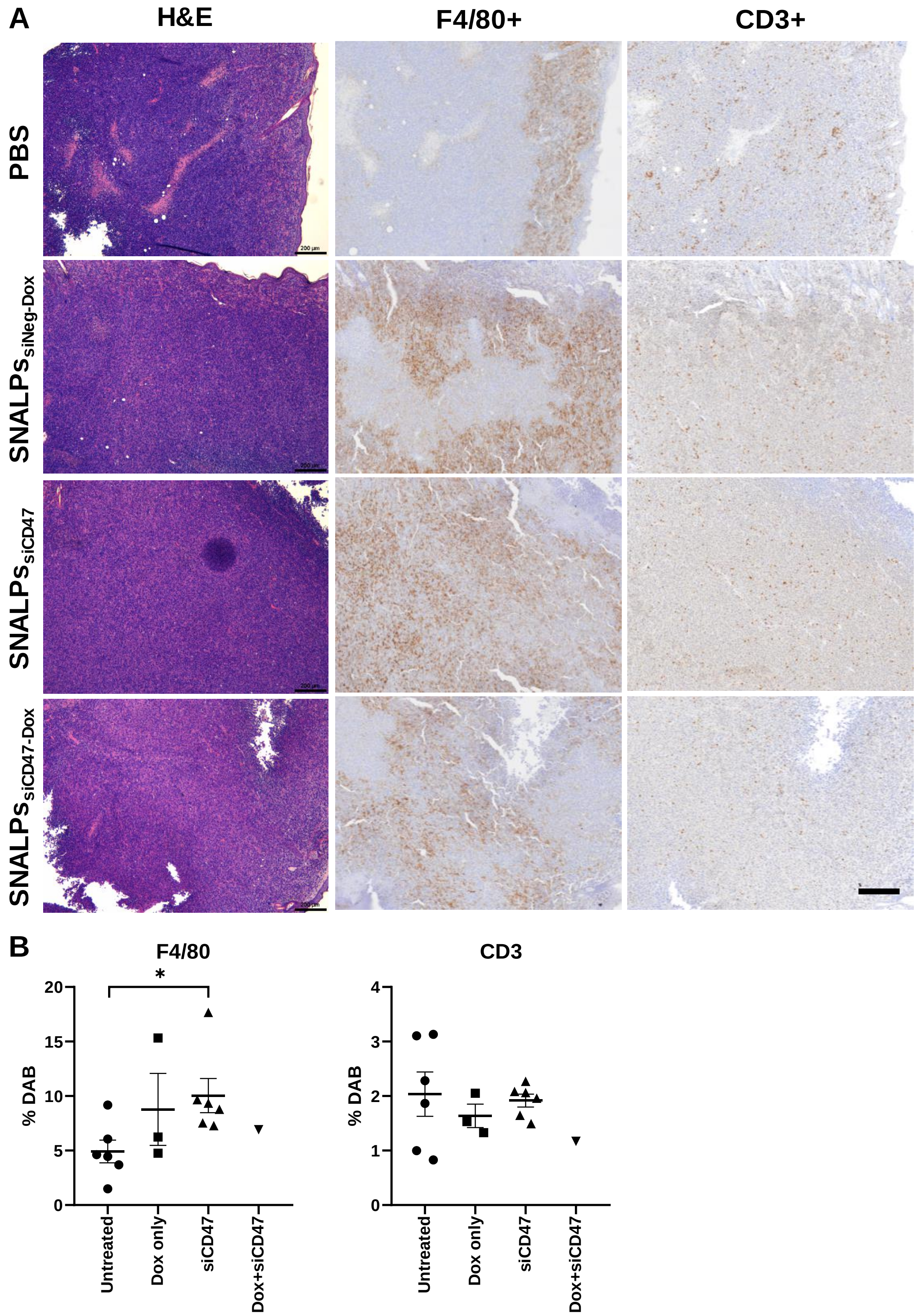

Figure S9. SNALPs$_{siNeg\text{-}Dox}$, SNALPs$_{siCD47}$, and SNALPs$_{siCD47\text{-}Dox}$ treatments increased macrophage infiltration in tumours. CT26 tumours from mice received PBS, SNALPs$_{siNeg\text{-}Dox}$, SNALPs$_{siCD47}$ or SNALPs$_{siCD47\text{-}Dox}$. Dox treatments were harvested after therapy studies. H&E and immunohistochemistry staining of F4/80 (macrophages) and CD3 (T cells) in tumours is demonstrated (**A**). Percentages of F4/80+ or CD3+ area per tumour area of the 4 groups are presented in (**B**). Scale bar, 200 μm. *p = 0.02. The H&E and IHC of F4/80+ and CD3+ images of the SNALPs$_{siCD47\text{-}Dox}$ treatment group was from a single available tumour sample that was histologically assessable. The data of % positive staining regions is shown in Fig S8B but not included in further statistical analysis.

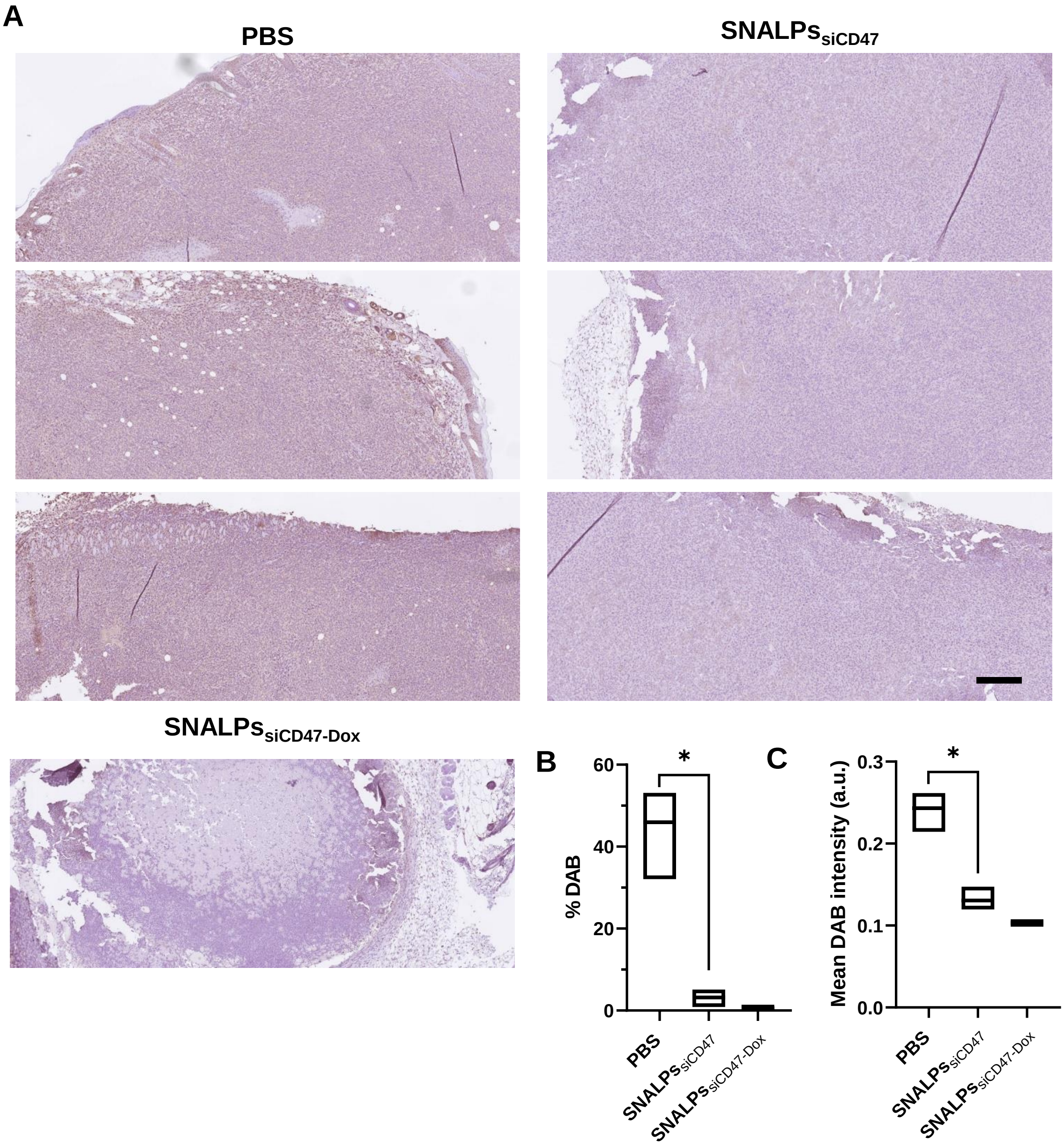

Figure S10. Expression of CD47 post SNALPs$_{siCD47}$ and SNALPs$_{siCD47\text{-}Dox}$ therapy examined by immunohistochemical analysis. CT26 tumours from mice received two doses of PBS, SNALPs$_{siCD47}$ or SNALPs$_{siCD47\text{-}Dox}$ on days 7 and 17 post tumour inoculation. Tumour tissues were harvested at the end of therapy studies on day 32 and proceeded to immunohistochemistry staining of CD47. Representative images of tumour tissues are demonstrated in **(A)**. Percentage of CD47 area (%DAB) and mean DAB intensity analyses are shown in **(B)** and **(C)**, respectively. *p = 0.02. Scale bar, 200 µm. The H&E and IHC of CD47 expression images of the SNALPs$_{siCD47\text{-}Dox}$ treatment group was from a single available tumour sample that was histologically assessable. The data of % positive staining regions is shown in Fig S10B and C but not included in further statistical analysis.
